# The effect of flooding on low birthweight and preterm birth: a systematic review and meta-analysis

**DOI:** 10.1186/s12889-026-26521-2

**Published:** 2026-03-05

**Authors:** Antonia Mendrinos, Elly Loyd, Meredith Jagger, C. Cozette Comer, Julia M. Gohlke

**Affiliations:** 1 Department of Population Health Sciences, Virginia Tech, Blacksburg, VA 24060 USA; 2 Independent Consultant, Austin, TX 78746 USA; 3 Evidence Synthesis Services, University Libraries, Virginia Tech, Blacksburg, VA 24060 USA

**Keywords:** Pregnancy, Preterm birth, Low birthweight, Floods, Tropical cyclone, Disaster, Systematic review, Meta-analysis

## Abstract

**Background:**

Numerous studies have examined pregnancy outcomes following flood events, with the majority focusing on two related outcomes: preterm birth (PTB) and low birthweight (LBW). Summarizing the results of these previous studies and determining remaining data gaps is the main objective of this systematic review and meta-analysis.

**Methods:**

We included publications in English that examined birthweight and/or gestational length related to exposure to floods, or events typically causing flooding (e.g. tropical cyclones). Seven academic databases were searched: CAB Abstracts (CABI), Academic Search Complete and Environment Complete (EBSCOhost), Environmental Science Index & Database (ProQuest), PubMed, Scopus, and Web of Science Core Collection. Searches were updated on February 23, 2025. For inclusion in meta-analyses, quantitative estimates of effect size and variance were required, and quality was assessed using the Quality Assessment Tool for Observational Cohort and Cross-Sectional Studies. Random effects regression was used for meta-analyses, and results are presented in forest plots, with potential for publication bias assessed in funnel plots and Egger’s test results.

**Results:**

Overall, data from 34 studies were extracted, and 25 studies across 13 countries were included in meta-analyses. Most studies (*N* = 18) examined tropical cyclone exposure. Meta-analyses indicate increases in LBW (RR = 1.03, 95% CI: 1.00, 1.05) and PTB (RR = 1.10, 95% CI: 1.00, 1.22). The LBW result was not significantly influenced by quality rating, while the PTB result is non-significant when all studies, regardless of quality rating, were included in the meta-analysis (RR = 1.01, 95% CI: 0.97, 1.05). Additionally, the PTB estimate is strongly influenced by one study with a large and highly significant effect size. Additional sub-analyses suggest no decreasing effect following more recent events (after 2005).

**Conclusions:**

Results are limited by the range of methods used across studies to estimate exposure to flooding and potential co-exposures related to events that caused the flooding (e.g. wind damage-related health outcomes during tropical cyclones). Regardless, results indicate that adverse pregnancy outcomes may increase following *in utero* exposure to flood events. Future studies incorporating finer spatiotemporally resolved estimates of exposure to flooding will improve estimates of effect. The study is registered in PROSPERO (CRD42024514540).

**Supplementary Information:**

The online version contains supplementary material available at 10.1186/s12889-026-26521-2.

## Background

Floods, the most common natural disaster, have been shown to have a wide range of impacts on human health, both acute and chronic [1]. Acute effects of floods include injuries, water-borne and food-borne illness from contaminated drinking water, and psychological distress [2]. Chronic or longer-term effects of flooding include infected wounds or other complications of injury, poor mental health, increases in vector-borne and other communicable diseases, and malnutrition [3]. Extreme flooding caused by hurricanes has been shown to increase release of chemical contaminants, such as lead and other harmful heavy metals [4]. Displacement from flooding also contributes to disruptions of social support networks and access to critical resources (e.g. medications and care) [5].

Previous research has characterized the effects of flooding events on general populations; however, research addressing the effect of floods on specific groups is limited. Factors such as gender, age, previous flood exposure, flood depth, socioeconomic status, and education have been associated with vulnerability during flood events [6, 7]. Stressful experiences, directly related to flooding or other traumatic events, have been shown to release cortisol and other stress-related hormones, negatively influencing pregnancy outcomes [8]. Studies have shown that pregnant women and newborns may be particularly vulnerable during flood events [9]. This may be due to disruption of prenatal care, evacuation and displacement, scarcity of essential resources, and psychological and physiological stress. Pregnant women face challenges during flood events, including unsafe labor and delivery conditions and unknown exposure to harmful toxins or toxicants [10, 11]. Complications from flood exposure may increase the risk of preterm birth, low birthweight, small for gestational age, still birth, and spontaneous abortion [9].

Due to climate change, catastrophic floods have increased in frequency, highlighting the need to better understand their impact on health, particularly among vulnerable populations [12]. By synthesizing existing evidence and performing a meta-analysis, this review aims to clarify the impact of flood-related events on perinatal health outcomes. Previous reviews have assessed the effect of disasters and stress on prenatal health; however, there is limited research looking at events specific to flooding [13]. Existing studies have investigated morbidity related to flooding, mainly for broader health outcomes, such as injury, infectious diseases, mental health, and adverse reproductive health outcomes [14–16]. Previous reviews that investigate adverse birth outcomes, including preterm birth, low birthweight, small for gestational age, spontaneous abortion, and stillbirth, have found mixed epidemiological evidence [17, 18]. To our knowledge, a meta-analysis focused on the effects of flooding on specific fetal outcomes such as preterm birth and low birthweight has not yet been conducted. Understanding the effect of floods on perinatal health is crucial for public health planning as improved prenatal care could help mitigate long-term adverse health outcomes associated with preterm birth and low birthweight [19]. Ultimately, we aim to guide future research directions and support community-level interventions.

Our objective for this systematic review and meta-analysis is to evaluate the effect of exposure to a flooding event during pregnancy on the perinatal health outcomes of preterm birth and low birthweight. Secondarily, we consider whether different types of flood-related events (e.g. tropical cyclones versus riverine or other flood events) affect preterm birth and low birthweight.

## Methods

### Protocols

This review was guided by the Campbell Collaboration’s Methodological Expectations (MECCIR) and a protocol was registered at PROSPERO with the registration number: CRD42024514540 [20, 21]. This manuscript was written in accordance with the Preferred Reporting Items for Systematic Reviews and Meta-Analysis (PRISMA) [22].

### Eligibility criteria

#### Inclusion criteria

Studies were eligible for inclusion if they met the following criteria:

1. The population studied was women who were pregnant during exposure to a flood related event;
2. Birthweight and/or gestational length, including stillbirth, was a reported outcome;
3. Quantitative exposure-response associations were available for our population of interest OR it must have been possible for us to disaggregate results of interest from aggregate results;
4. Studies were primary, observational with either cohort, case-control, cross-sectional, time series, or case-crossover designs;
5. Studies must be available in English.

Studies of our population of interest that also considered other populations were included. We defined flood-related events as any event described directly as a flood, or weather events in which flooding typically occurs (e.g. hurricane, cyclone). Studies in which other events were examined (e.g. earthquakes, a dam broke) *and* flooding was documented as a primary exposure (mentioned in title or abstract) were also included. Records were included if mediators or moderators were the primary focus of the paper, but the exposure and outcome was relevant. Preterm birth was defined as birth before 37 weeks gestation [23]. Low birthweight was defined as weight at birth below 2500 g [24]. We did not limit studies by date of publication or data collection.

### Exclusion criteria

Studies were excluded if the study met any of the following criteria:

1. Wrong Study Design – Used for reviews, editorials, etc.;
2. Wrong Patient Population – Used when the population is someone other than pregnant women exposed to a flood event;
3. Wrong Outcome – Used when no quantitative measure of birthweight or gestational length is reported;
4. Wrong Exposure – Used when flooding may not be the primary exposure and/or could contribute to confounding; for example, studies focused on populations in the Fukushima Prefecture after the 2011 tsunami from the Great East Japan Earthquake were excluded.

### Search strategy

Studies were located via a comprehensive search of the following academic databases: PubMed (NNLM), Environment Complete (EBSCOhost), CAB Abstracts (CABI), Environmental Science Index & Database (ProQuest), Academic Search Complete (EBSCOhost), Scopus, and Web of Science Core Collection. Initial searches were run on April 4, 2024, and searches were rerun on February 23, 2025. Searches were conducted by an expert searcher (CC). The full search strategy is reported according to PRISMA-S (See Additional File 1) [25].

After the full text review, the citation chasing tool *citationchaser* was used to find work cited in included records and relevant reviews. This process was also used to identify references that had since cited those records [26]. Results were deduplicated against records already found through the comprehensive search.

### Study selection

The team used the systematic review software, Covidence [27], to manage the screening process. Duplicate records were removed automatically when uploaded into Covidence. All titles and abstracts of unique records from the search were screened by two independent reviewers. Three of the authors served as reviewers (AM, EL, MJ) and one author was involved in discussions toward resolving conflicts (JG). Interrater reliability (IRR) was calculated automatically in Covidence. For each pairing of reviewers, Covidence calculates the IRR using final decisions from each reviewer. In the title and abstract screening phase, ‘maybe’ votes were counted as ‘yes’ votes. Both basic proportion of agreement and Cohen’s Kappa are calculated. Across the pairs of reviewers, the team’s Cohen’s Kappa score for title and abstract screening ranged between 0.53 and 0.82 (See Additional File 3 3).

Full text was retrieved via the automated full text finders in Zotero and EndNote. The rest of the articles were sought manually, for example, by searching Google Scholar. Remaining studies that could not be located were retrieved through Virginia Tech’s Interlibrary Loan system. Dual independent screening of all full text records was conducted by four of the authors (AM, EL, JG, MJ). Across reviewer pairings, Cohen’s Kappa ranged from 0.37 to 1.00 (See Additional File 3).

Two independent reviewers also screened results from citation chasing and the search update in a spreadsheet (Google Sheets) outside of Covidence (AM, EL, JG, MJ). Records that were identified as possibly relevant were uploaded into Covidence for full text screening and data extraction. Any disagreements throughout this process were resolved by discussion with at least three of the five authors (AM, CC, EL, JG, MJ).

### Data extraction

The following data were extracted from the relevant studies and/or their supplemental material: exposure, measurement of exposure (i.e. geospatial, temporal, geography, Federal Emergency Management Agency (FEMA) declaration, other government aid, wind (Saffir-Simpson Hurricane Intensity Scale), frequency/quantity, rainfall intensity, social support questionnaire, displacement, or stress), population characteristics, year(s) of study, geographical region, outcomes studied, statistical analysis performed, covariates, methodology, and results. For each outcome, we extracted the exposure(s), measure, measured result, lower and upper confidence interval (CI), sample size, trimester specific exposure (if reported), and other demographic sub-analysis. Using Covidence, two independent authors (AM, EL, JG, MJ) extracted results from each study. A third independent author (AM, EL, JG, MJ) reviewed the extracted data to select a final decision and resolve conflicts. Disagreement was resolved through discussion with all five authors.

### Risk of bias (quality) assessment

To assess risk of bias in included studies, we used the Quality Assessment Tool for Observational Cohort and Cross-Sectional Studies from the National Institute of Health Heart, Lung, and Blood Institute [28]. We supplemented this tool with an additional four questions covering reliability, validity, and reproducibility from the Public Health Ontario MetaQAT critical appraisal tool [29]. Domains considered in the assessment included risk of potential for selection, information, or measurement bias, or confounding via evaluation of study design components (exposure measurement prior to outcomes, dose-response, accuracy of measurement of exposure and outcome, time lags between exposure and effect, and controls for confounding). Risk of bias assessments were completed by the same two investigators that independently completed data extraction and included supporting text for judgements for each domain. Investigators assigned an overall quality judgement based on responses to the 18 elements. As with data extraction, a third investigator reviewed the two responses, and conflicting responses were discussed to determine the final consensus overall quality judgement of “Good”, “Fair”, or “Poor.” Consensus responses for each of the 18 elements of the quality assessment, as well as the final consensus overall quality judgement and justification, are reported in Additional File 2, Table S1, Columns ADM-AEX.

### Synthesis

We performed random effects meta-analyses of studies reporting preterm birth (PTB) and low birthweight (LBW) outcomes. Other outcomes were not analyzed due to small sample size.

### Methods for exposure selection

For studies included in our meta-analyses, we selected the results from each study’s final, adjusted model. Across included studies, the specific measurement and definition of exposure to flooding events, weather events in which flooding typically occurs, or other events where flooding was documented as a primary exposure, varied. We selected the result using the following criteria:

1. Exposure to any event: Many studies reported on one event. In cases where a study included multiple events, such as all hurricanes in a season or multiple years of flooding, we included the overall result for exposure to “any event,” rather than selecting one of the individually reported events.
2. Exposure any time during pregnancy: Most studies reported on exposure any time during pregnancy. When available, the overall result was included, rather than trimester-specific results. When only trimester-specific results were reported, we consistently selected results for exposure during the third trimester because of the temporal connection to PTB.
3. Geographic proximity: When results for multiple geographic areas were reported, for example state and city, or regions based on level of disaster declared, the most impacted geography was selected; that is, results from a city where flooding was most severe were included rather than the state-wide results, or if results from multiple levels of disaster assistance were reported, the highest level was included.
4. Meteorological definition of exposure: In cases where multiple meteorological measurements for tropical cyclone exposure were reported, we consistently selected the measurement closest to hurricane-force winds (i.e. winds of 74 mph or greater, or Saffir-Simpson category).

Standard transformations were completed to convert reported results into comparable effect estimates [30]. Reported odds ratios and percent increases (absolute or relative) were converted to estimated rate ratios, and reported confidence intervals and *p*-values to standard errors using methods described in the Cochrane Handbook for Systematic Reviews of Interventions, Chaps. 6, 14 and 15 [30]. Table S3 in Additional File 2 provides equations used and the specific reference for each transformed effect estimate for each included study. The DerSimonian-Laird estimator was used to estimate between-study variance. Forest plots were generated to present results for both outcomes (PTB, LBW) separately.

Meta-analyses were performed with our main analyses only considering studies with low risk of bias, rated “Good” in risk of bias (quality) assessment. We also conducted sub-analyses on studies examining events through 2005 versus studies examining events occurring after 2005, to determine if temporal trends in association are evident. Another subgroup analysis included studies examining the effects of flooding from tropical cyclones as the exposure (excluding those studies examining flooding from other sources), since there is an expectation that other tropical cyclone-related factors (e.g. wind damage) could influence the relationship with health outcomes. Additional subgroup analyses included examination of studies adjusting for maternal age and maternal education, since these were the two most common covariates included in models, and a subgroup analysis of studies conducted on events happening in the United States. Additional country specific subgroup analyses were not possible, due to low sample size. All analyses were performed in R using the Meta package [31] and the script used is provided in Additional File 4.

### Reporting bias assessment

Funnel plot asymmetry was evaluated using the Egger’s test to assess potential for publication bias using the Meta package in R [32].

## Results

### Study selection

**Fig. 1:**
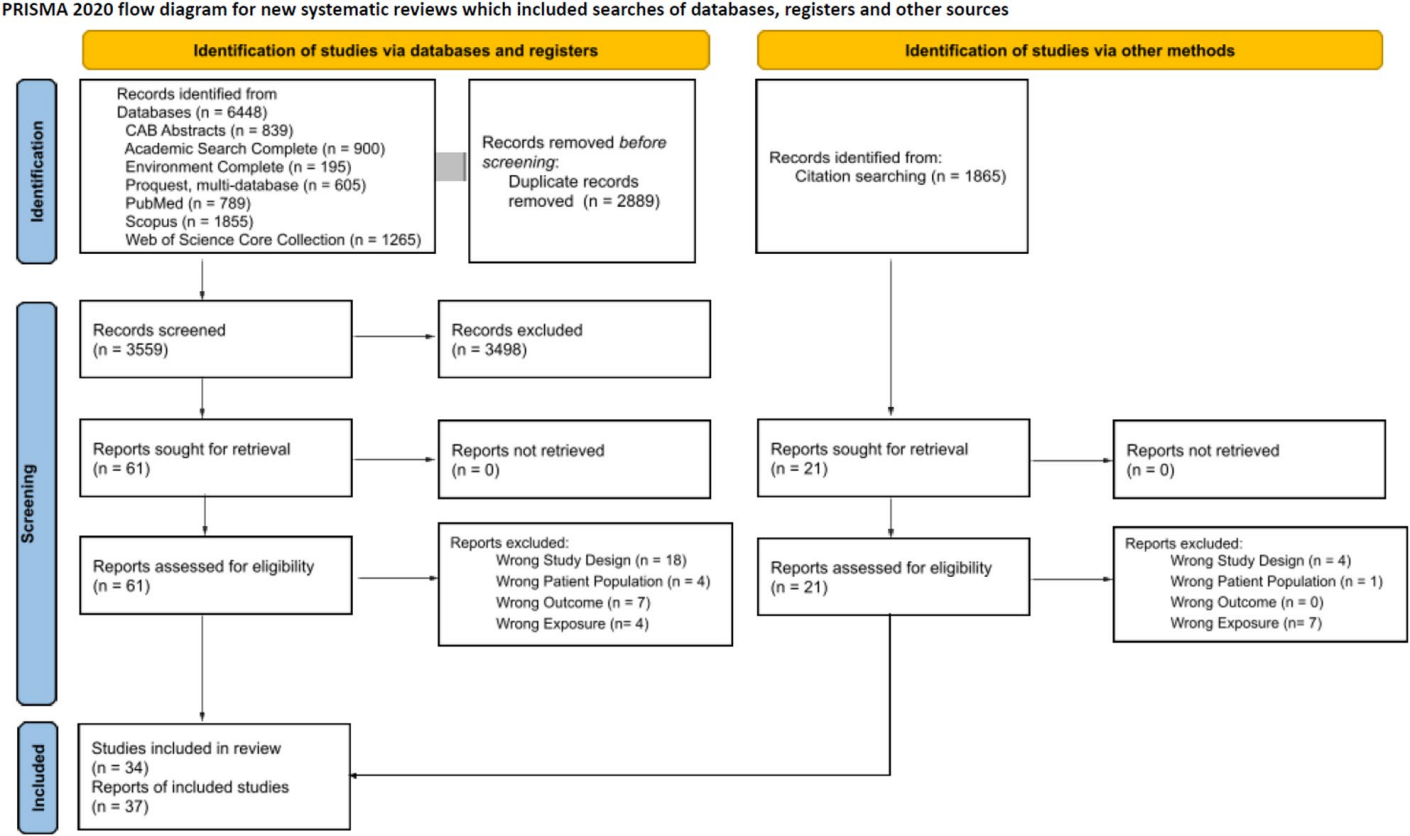
PRISMA Flow Diagram

The initial search yielded a total of 5,898 records and 3,195 after deduplication. The search update returned 550 new records and 364 unique records after deduplication. Results from the database searches in Fig. 1 show the total yield across both the initial search and search update. During title and abstract screening, 3,498 records were identified as irrelevant. The full text of 61 records were assessed for eligibility and 28 records were included (Fig. 1). We identified an additional 1,865 unique records from citation chasing resulting in 21 additional publications identified for full text review. Following the same dual independent reviewer process, 9 publications were identified for data extraction. In total, 34 studies (37 reports) were identified as relevant.

### Study characteristics

Of the 34 studies from which data were extracted, 25 studies were included in the meta-analysis for either LBW and PTB, 14 reported on both outcomes, six only reported LBW, and five only reported PTB (Table 1). Eight studies were excluded from the meta-analysis because the outcome measure was gestational age or birthweight as a continuous variable, and authors did not include analysis of the dichotomized outcomes of PTB or LBW (Table 1). One study was excluded because no estimate of variation (SE or 95% CIs) was reported (Table 1).

**Table 1:** Study characteristics

First Author, Publication Year [Reference]	Exposure(s)			Exposure Description	Year(s) of Event(s)	Year(s) of Health Data	Outcome(s)							Sample Size	Location
	F	TC	O				BW	GA		LBW		PTB			
Included in Meta-analysis															
Antipova, 2015 [33]		x		Single event	1992	1991–1995					x		x	12,349	USA
Beuermann, 2017 [34]		x		Multiple events, multiple years	1987–2014	1993–2012	x				x			1,764	Jamaica
Biswas, 2024 [35]			x	Zone mapping	2019–2021	2019–2021					x			202,194	India
Chacón-Montalván, 2021 [36]	x			Multiple events, multiple years	2006–2017	2006–2017	x				x		x	291,478	Brazil
Chen, 2012 [37]		x		Single event	2005	2003–2006					x		x	1,467	USA
Currie, 2013 [38]		x		Multiple events, multiple years	1996–2008	1996–2008			x		x			485,048	USA
de Oliveira, 2023 [39]		x		Single event	2004	2001–2005	x				x		x	53,006	Brazil
Grabich, 2016 [40]		x		Multiple events, single year	2004	2003–2004					x		x	67	USA
Grabich, 2016 [41]		x		Multiple events, single year	2004	2003–2005							x	41,025	USA
Grabich, 2017 [42]		x		Multiple events, single year	2004	2003–2004					x			67	USA
Harville, 2010 [43]		x		Single event	2005	2003–2007					x		x	46,242	USA
Harville, 2023 [44]		x		Single event	2018	2017–2019					x		x	13,265 (PTB), 14,097 (LBW)	USA
Hetherington, 2021 [45]	x			Single event	2013	2012–2015							x	18,291	Canada
Hochard, 2022 [46]		x		Single event	2011	2006–2012	x		x		x		x	710,310	USA
Liu, 2024 [47]		x		Single event	2017	2015–2019							x	15,564	USA
Nasir, 2018 [48]			x	Multiple events, multiple years	2010–2011	2010–2011	x				x			737	Pakistan
Parayiwa, 2022 [49]		x		Multiple events, multiple years	2011, 2015, 2017	2008–2018					x		x	101,942	Australia
Spurlock, 2025 [50]		x		Single event	2003	2000–2009					x		x	62,978	USA
Sugawara, 2018 [51]			x	Single event	2011	2011					x		x	12,808	Japan
Sugg, 2023 [52]	x	x		Single event	2015	2013–2016					x		x	74,154	USA
Sun, 2020 [53]		x		Multiple events, multiple years	1989–2002	1989–2002							x	1,963	USA
Tong, 2011 [54]	x			Single event	1997	1994–2000					x		x	21,520	USA
Xiong, 2008 [55]		x		Single event	2005	2006–2007					x		x	220	USA
Yang, 2024 [56]	x			Multiple events, multiple years	1995–2020	1995–2020	x						x	1,338,314	Australia
Yu, 2018 [57]	x	x	x	Multiple events, multiple years	1994–2012	1994–2012							x	458,820	USA
Excluded From Meta-analysis															
Baloch, 2012 [58]	x			Single event	2010	2010	x							571	Pakistan
Hamilton, 2009* [59]		x		Single event	2005	2004–2006					x		x	166,675	USA
Hilmert, 2016 [60]	x			Single event	2009	2009	x		x					136	USA
Ishikuro, 2022 [61]			x	Single event	2011	2009–2014	x		x					925	Japan
Kroska, 2018 [62]	x			Single event	2008	2008	x							103	USA
Le, 2022 [63]			x	Multiple events, multiple years	2004–2011	2002–2012	x							1,961	Vietnam
Morrow, 2014 [64]		x		Multiple events, multiple years	1989–2008	1989–2008	x							6,305	Philippines
Nguyen, 2023 [65]			x	Multiple events, multiple years	2007–2012	2007–2012	x							1,954	Kyrgyzstan
Sanguanklin, 2014 [66]	x	x	x	Multiple events, multiple years	2011–2012	2011–2012	x		x					175	Thailand

*Abbreviations*: *F* flood, inland, riverine, *TC* tropical cyclone, hurricane, typhoon, *O* other (i.e. monsoon, tsunami, direct measurement of precipitation), *BW* birthweight, *GA* gestational age, *LBW* low birthweight, *PTB* preterm birth. Except where noted, all studies excluded from meta-analysis lacked PTB or LWB outcome. *Excluded because reported numerical results could not be transformed

**Fig. 2:**
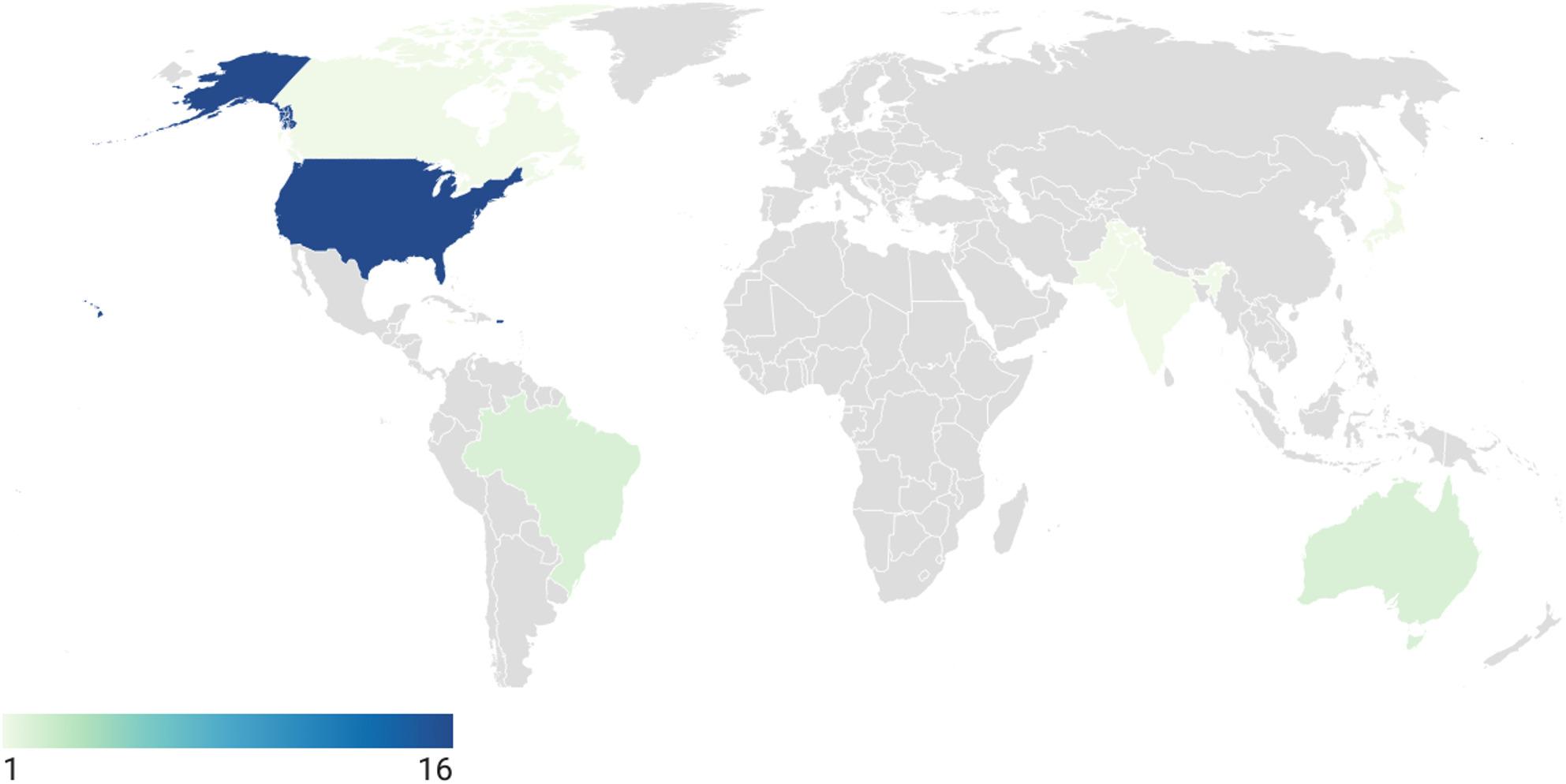
Map of countries in which LBW or PTB has been evaluated following flooding events. Color indicates the number of studies. All studies (*N* = 25) included in the meta-analyses are represented, and further detail on study characteristics are provided in Table 1. Map created in app.datawrapper.de

The spatial and temporal resolution of the health data dictated the types of exposure data that could be evaluated. For example, in some studies, aggregate county-level, administrative birth records were used, and therefore a county-level measure such binary hurricane exposure was selected. In other cases, addresses from individual health records could be more precisely matched to flooding measures. Most of the studies included in the meta-analyses (*N* = 18) evaluated tropical cyclone (hurricane or typhoon) exposure; six evaluated riverine or inland flooding; and four included another event with flooding, including monsoon, tsunami, and direct measurement of precipitation. Exposures are not mutually exclusive. Thirteen of the studies quantified outcomes for a single event, three focused on multiple events in a single year, eight included multiple events over multiple years, and one used flood zone mapping. Studies evaluated impacts across 12 countries (Table 1; Fig. 2), with 16 of the 25 studies included in the meta-analysis evaluating events that occurred in the United States.

### Risk of bias in studies

Considerations of spatial and temporal resolution in health outcome and exposure measurement, as well as suitability of statistical methods, were primary considerations for final composite quality ratings (Table 2). Assessment responses are detailed for individual components of the risk of bias (quality) assessment tool for each study that was extracted (See Supplemental Table S1, Additional File 2).

**Table 2:** Quality rating, exposure, and effect estimates for studies included in the meta-analyses

First Author, Publication Year [Reference]	Quality Rating	Selected Exposure	LBW Effect Estimate (SE)	PTB Effect Estimate (SE)
Hazard Ratio				
Grabich, 2016* [41]	Good	County-level wind speed greater than or equal to 74 mph during hurricane	NR	1.06 (0.03)
Yang, 2024* [56]	Good	Flood exposure (third trimester)	NR	0.62 (0.01)
Odds Ratio				
Antipova, 2015 [33]	Good	20-mile buffer overlaid on all parishes declared as disaster areas	0.97 (0.05)	1.25 (0.07)
Chacón-Montalván, 2021 [36]	Good	Intense exposure to extreme rain events	1.57 (0.60)	8.78 (0.84)
Harville, 2010 [43]	Fair	Parishes most severely affected by hurricane	0.96 (0.03)	0.87 (0.02)
Liu, 2024 [47]	Fair	1-week lag exposure to hurricane	NR	1.05 (0.08)
Parayiwa, 2022 [49]	Good	Any cyclone (late pregnancy)	0.97 (0.08)	0.99 (0.06)
Sugawara, 2018 [51]	Fair	Coastal facilities compared to inland facilities	0.92 (0.10)	0.85 (0.28)
Tong, 2011 [54]	Poor	Counties most severely affected by flood	1.17 (0.08)	1.07 (0.05)
Xiong, 2008 [55]	Fair	Three or more of eight severe hurricane experiences	2.61 (0.96)	1.89 (0.65)
Yu, 2018 [57]	Poor	Annual flood data	NR	1.02 (0.001)
Rate Ratio				
Beuermann, 2017 [34]	Good	Coastal-rural area when hit by at most one hurricane (third trimester)	0.97 (0.15)	NR
Biswas, 2024* [35]	Good	Flood prone areas compared to non-flood prone areas	1.08 (0.02)	NR
Chen, 2012 [37]	Fair	African American women, comparing Gulf Coast states to other U.S. states after hurricane	1.77 (0.54)	1.49 (0.45)
Currie, 2013 [38]	Good	Within 30 km one of eight tropical storms or hurricanes	1.00 (0.01)	NR
de Oliveira, 2023 [39]	Good	Municipalities within 200 km of hurricane	3.10 (4.80)	1.21 (1.76)
Grabich, 2016 [40]	Fair	County-level severity of wind speed based on Saffir-Simpson categories	0.71 (0.30)	0.61 (0.20)
Grabich, 2017 [42]	Fair	County-level wind speed greater than or equal to 74 mph during hurricane season	0.73 (0.71)	NR
Harville, 2023* [44]	Good	Counties receiving both public and individual assistance post-hurricane	1.19 (0.06)	0.96 (0.04)
Hetherington, 2021 [45]	Good	Living in flood area	NR	1.00 (0.01)
Hochard, 2022 [46]	Good	In state during hurricane	1.01 (0.002)	1.01 (0.002)
Nasir, 2018 [48]	Poor	District-level exposure to flood	0.90 (0.45)	NR
Spurlock, 2025* [50]	Good	30-km buffer from hurricane	0.74 (0.11)	0.71 (0.08)
Sugg, 2023* [52]	Good	FEMA-designated counties for public assistance after extreme flooding	1.10 (0.09)	0.99 (0.08)
Sun, 2020* [53]	Good	County-level tropical cyclone-related peak sustained winds greater than 17.2 m/s	NR	1.01 (0.01)

Meta-analysis Results. Overall results, exposure of all persons at any time during pregnancy, are presented except where noted. When included, we extracted the final, adjusted model from each study. Transformed effect estimates are presented here. *Abbreviations*: *LBW* low birthweight, *PTB* preterm birth, *SE* standard error, *NR* not reported in this study. *Results from these studies were not transformed for meta-analysis. Groupings: Odds Ratio includes exponentiated logistic regression coefficient; Rate Ratio includes difference in difference (DID), difference in difference in difference (DDD), difference estimators, marginal mean difference, percent probability, percentage points. For full details on transformations, see Supplemental Table S3, Additional File 2

### Results of meta-analyses

The quality rating, exposure measurement, and effect estimates for studies included in the meta-analyses are presented in Table 2. Table 3 provides additional details on the exposure metric characteristics and covariates included for each of the studies. Most studies estimate rate ratios (14 of the 25 studies), although some calculated odds ratios or a hazard ratio (Table 2), based on the data available for outcome and exposure measurements (Table 3). The most common methods for exposure characterization included assignment of home locations to geographical units, such as administrative level 2 (ADM2), which corresponds to counties in the United States or ADM1 (states in the United States), and assignment of exposure based on a flood designation within that geographical unit during the time of gestation (Table 3). As detailed in Table 3, there are a range of flood estimations used, including government aid designations for flood victims, wind strength, tropical cyclone path, and rainfall intensity.

**Table 3:** Exposure, Case, and covariate details for studies included in the meta-analyses

First Author, Publication Year [Reference]	Measurement of Exposure									Case Ascertainment	Covariates															
	Geospatial	Temporal	Geographical Units	FEMA	Other Government Aid	Wind	Frequency/Quantity	Rainfall Intensity	Other		Sex (of infant)	Smoking	Income	Education	Maternal Age	Conception Year	Conception Month	Parity	Geographic Region	Environmental Quality Index	Nicotine Use	BMI	Marital Status	Race	Ethnicity	Other
Antipova, 2015 [33]	x	x							x	Individual administrative birth records														x		
Beuermann, 2017 [67]	x	x	x			x	x		x	Nationally representative household survey	x			x	x											x
Biswas, 2024 [68]			x						x	Nationally representative household survey	x			x					x							x
Chacón-Montalván, 2021 [36]	x	x						x		Individual administrative birth records	x			x	x	x	x		x				x	x	x	x
Chen, 2012 [37]		x	x							Aggregate administrative birth records																x
Currie, 2013 [34]	x	x				x				Individual administrative birth records				x	x	x	x		x				x	x		x
de Oliveira, 2023 [35]	x	x			x					Individual administrative birth records	x			x	x			x	x				x	x		x
Grabich, 2016 [40]	x	x		x		x				Aggregate administrative birth records										x						
Grabich, 2016 [41]	x	x	x			x	x			Individual administrative birth records				x	x						x		x	x	x	x
Grabich, 2017 [42]	x					x				Individual administrative birth records																
Harville, 2010 [43]		x	x							Individual administrative birth records		x		x				x					x	x	x	x
Harville, 2023 [44]		x	x	x						Individual administrative birth records		x		x	x							x			x	x
Hetherington, 2021 [38]	x	x								Individual administrative birth records					x											
Hochard, 2022 [46]	x	x	x					x	x	Individual administrative birth records						x	x		x							
Liu, 2024 [39]	x	x				x				Individual administrative birth records	x	x		x	x		x	x				x	x	x	x	x
Nasir, 2018 [45]		x	x		x				x	Nationally representative household survey	x		x	x	x			x	x							x
Parayiwa, 2022 [47]		x	x		x					Individual administrative birth records	x	x			x			x	x				x			x
Spurlock, 2025 [48]	x	x		x						Individual administrative birth records & prospective cohort survey				x	x									x	x	x
Sugawara, 2018 [49]		x	x							Individual facility birth records	x				x											x
Sugg, 2023 [52]	x	x		x						Individual administrative birth records		x		x	x									x	x	x
Sun, 2020 [53]	x	x				x		x		Individual administrative birth records																x
Tong, 2011 [54]		x	x							Aggregate administrative birth records		x		x	x								x	x	x	x
Xiong, 2008 [50]							x		x	Individual administrative birth records & prospective cohort survey		x	x	x	x			x					x			x
Yang, 2024 [51]	x	x	x				x			Individual administrative birth records	x	x			x											x
Yu, 2018 [55]		x	x					x		Individual administrative birth records																x

Meta-analysis Exposure, Case, and Covariate Details. For measurements of exposures, in our extraction form, "Geospatial" was defined as "spatial modeling to define exposure, distance" and was used most frequently in studies where a storm track and distance buffer were used. "Geography" was defined as "geographical units to define exposure, ex: regional, flood-prone areas" and was most often administrative geographies such as counties or states. "Wind" was defined as the "Saffir-Simpson Hurricane Intensity Scale." "Frequency/Quantity" was defined as "ex: exposed to 1,2,3 hurricanes." For details on the "Other" measurements of exposure and "Other" Covariates, see columns F and L respectively in S1.Extraction & Qual Assessment dataset presented in Additional File 2. Abbreviations: FEMA = Federal Emergency Management Agency; BMI = Body Mass Index

### Low birthweight

**Fig. 3:**
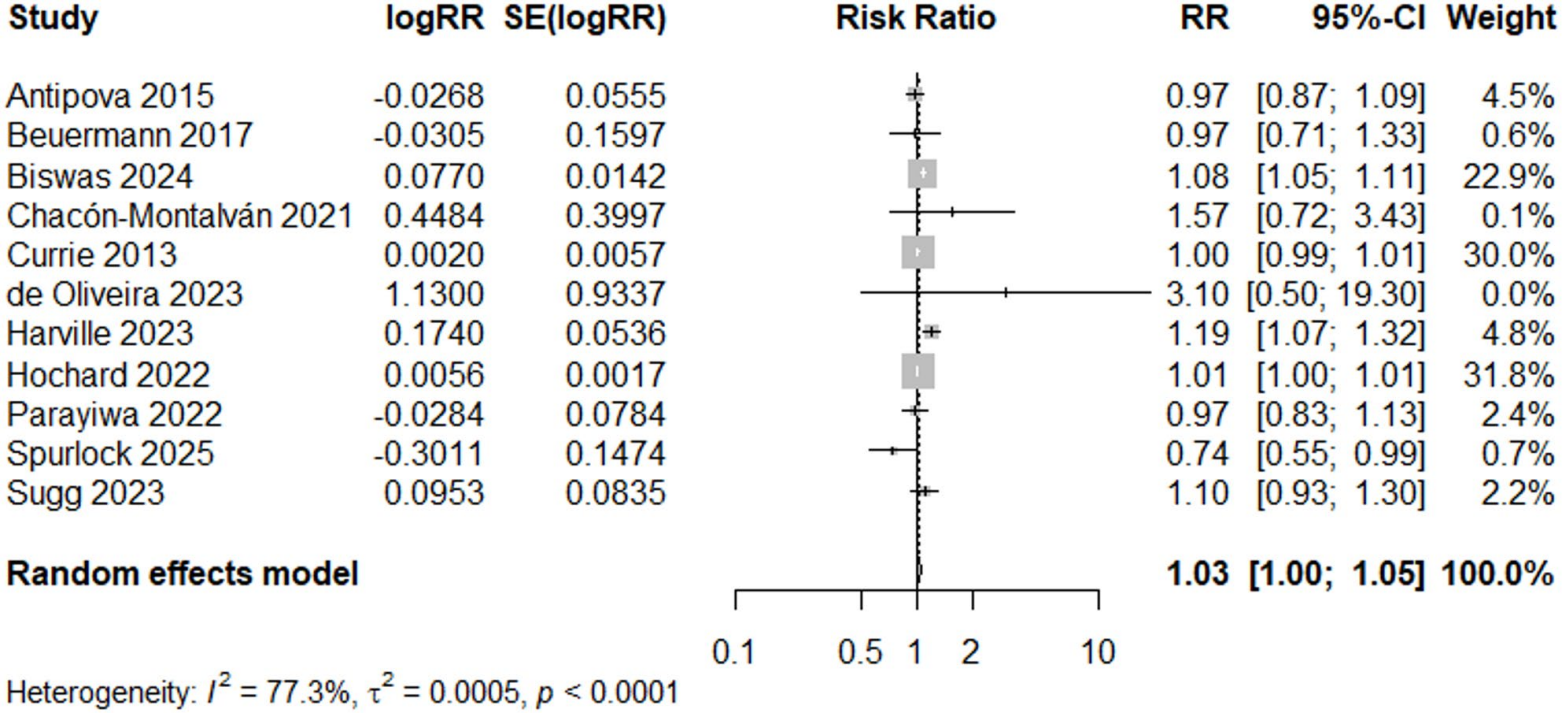
Forest Plot for LBW Studies, “Good” Quality Rating (*N* = 11)

**Fig. 4:**
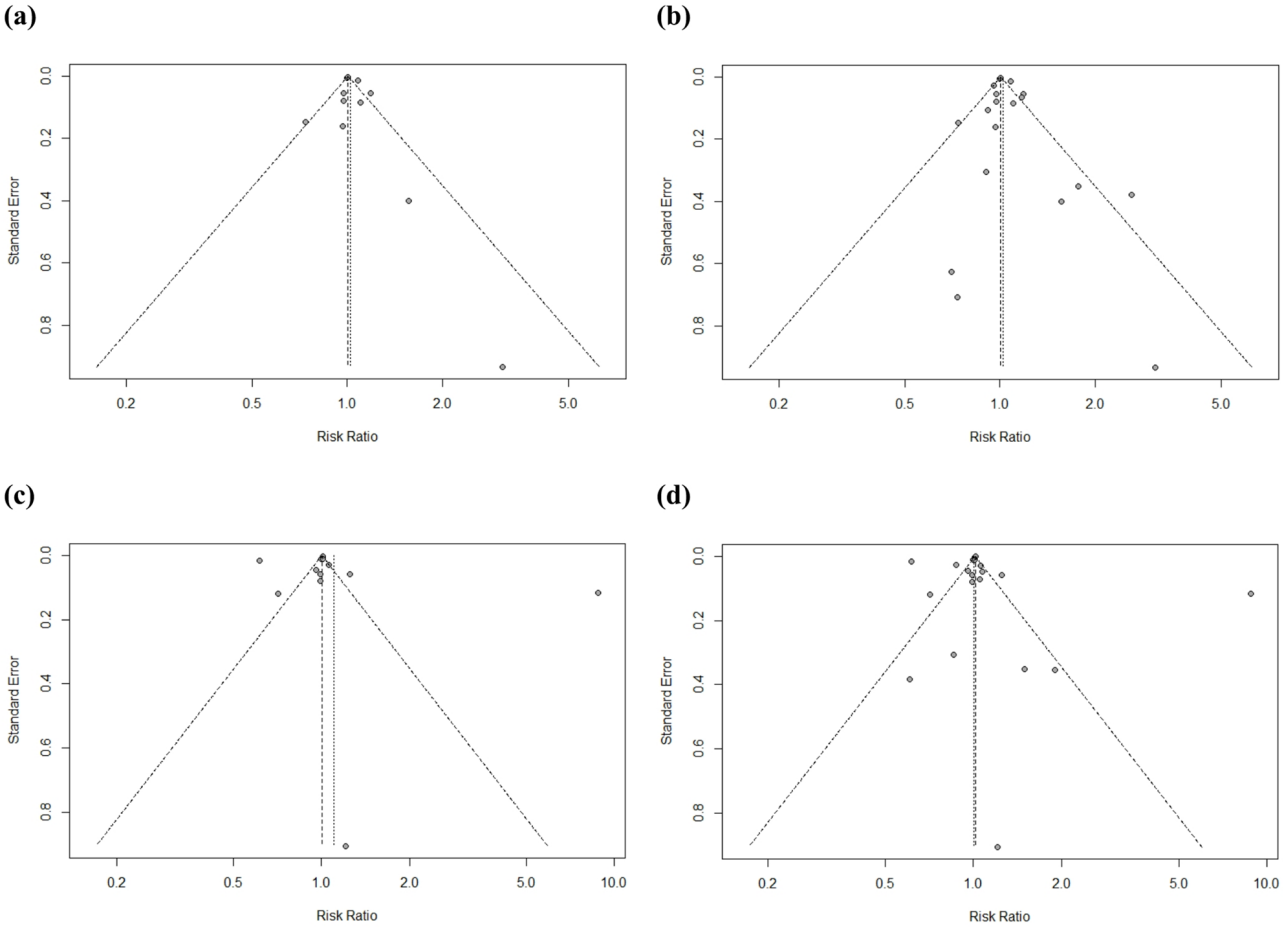
Funnel Plots (**a**) LBW Studies, “Good” Quality Rating (*N* = 11) (**b**) LBW Studies, Overall (*N* = 19) (**c**) PTB Studies, “Good” Quality Rating (*N* = 12) (**d**) PTB Studies, Overall (*N* = 20)

When filtering by study quality, the risk ratio for “Good” quality studies (*N* = 11) indicated a 3% increased risk of low birthweight (RR = 1.03, 95% CI: 1.00, 1.05) using a random effects model (Fig. 3). Visual inspection of the funnel plot showed a symmetrical distribution of studies, indicating no strong evidence of publication bias (Fig. 4a). This was further supported by Egger’s test [32] for funnel plot asymmetry (*p* = 0.29).

**Fig. 5:**
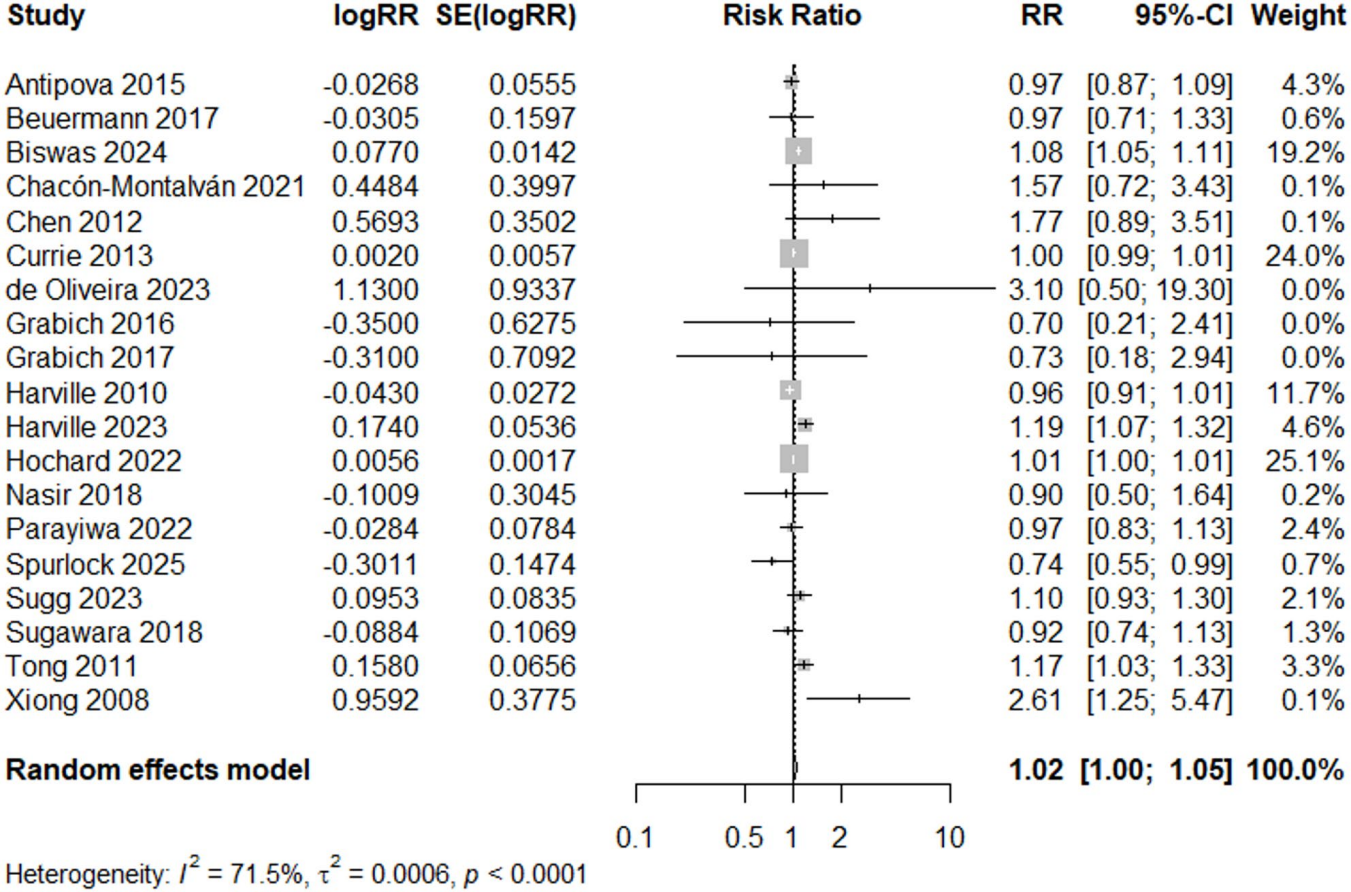
Forest Plot for LBW Studies, Overall (*N* = 19)

When all studies (*N* = 19) are included in the meta-analysis, regardless of study quality rating, the overall estimate remained similar (RR = 1.02, 95% CI: 1.00, 1.05) (Fig. 5), and there was no indication of bias based on funnel plot inspection (Fig. 4b) and Egger’s test (*p* = 0.23). Both results indicate high heterogeneity (I^2^ =77% and 71%, respectively), therefore sub-analyses were conducted.

**Fig. 6:**
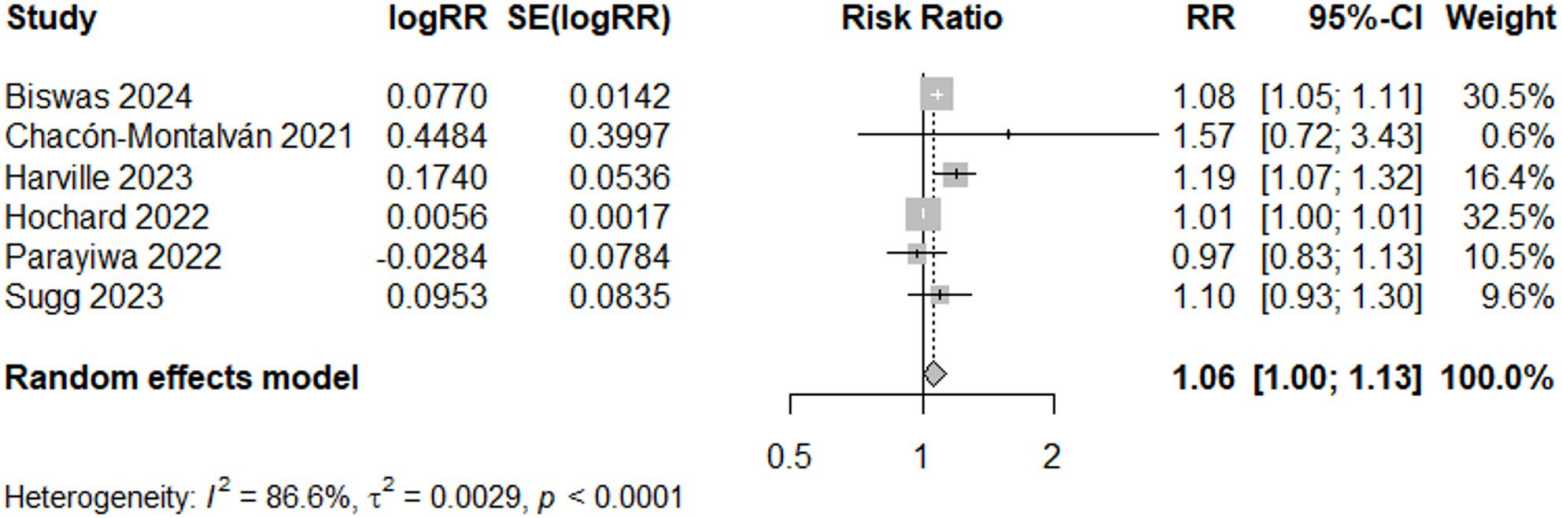
Forest Plot for LBW Studies, “Good” and Recent Event > 2005 (*N* = 6)

We applied a filter by event year to determine if results were affected by advances in public health infrastructure and disaster response. We chose the cutoff for “early” event years as studies with flooding preceding or during 2005. Most of the studies were conducted in the U.S., and Hurricane Katrina exposed major gaps in disaster response capacity and disproportionately affected vulnerable groups like pregnant women [69]; therefore, post-Katrina emergency management improvements could reduce adverse effects from flood events. In the sub-analysis of good quality studies with recent event years (after 2005) (*N* = 6), we observed a 6% increased risk of low birthweight (RR = 1.06, 95% CI: 1.00, 1.13) (Fig. 6). Egger’s test for publication bias was not significant (*p* = 0.13).

**Fig. 7:**
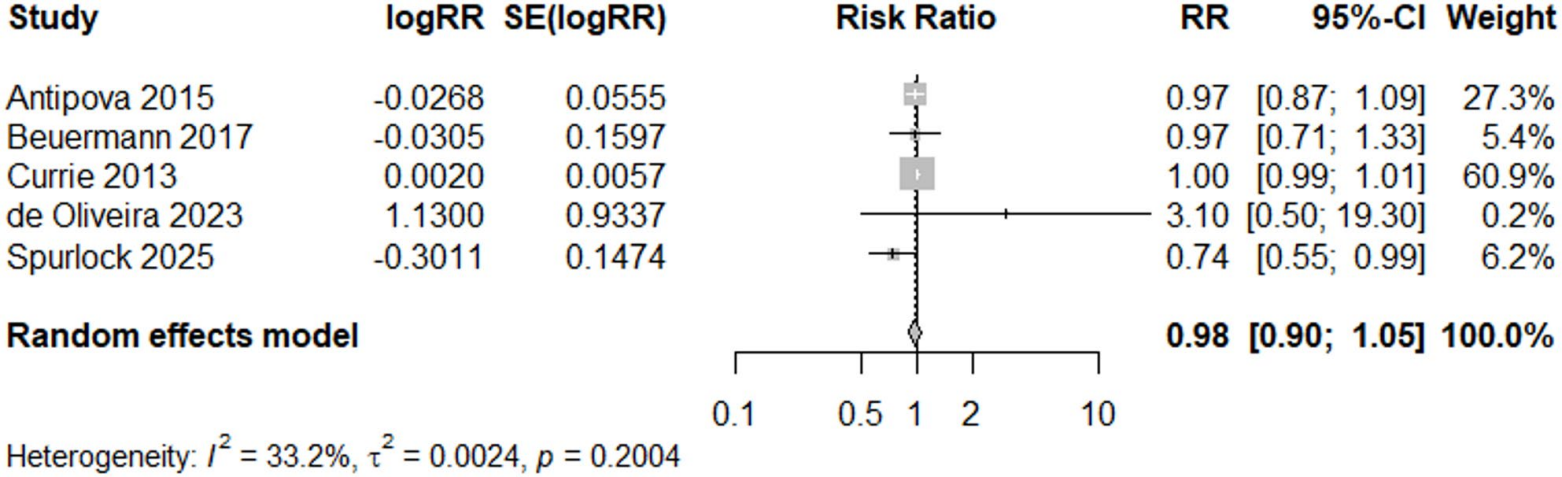
Forest Plot for LBW Studies, “Good” and Early Event ≤ 2005 (*N* = 5)

In contrast, among good quality studies with early event years (2005 or earlier) (*N* = 5), the risk ratio was 0.98 (95% CI: 0.90, 1.05) (Fig. 7), suggesting no change in low birthweight following flood events in early years. Heterogeneity was reduced and non-significant for the meta-analysis of events during and before 2005 (I^2^ = 33%), while heterogeneity remained high (I^2^ = 87%) in the meta-analysis of more recent events.

**Fig. 8:**
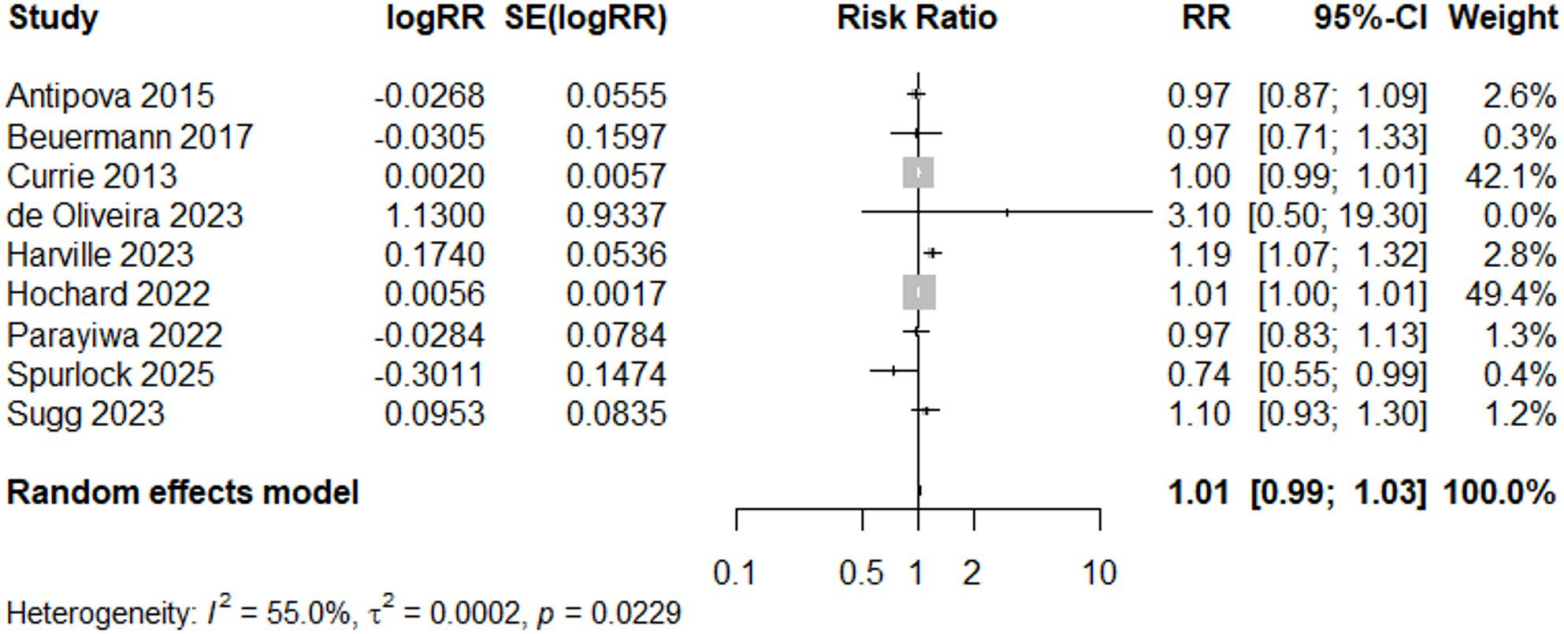
Forest Plot for LBW Studies, “Good” and TC-Related (*N* = 9)

We also conducted a sub-analysis only including tropical cyclone-related flooding. Meta-analysis of the 9 “Good” quality studies suggests a non-significant increase (RR = 1.01, 95% CI: 0.99, 1.03) in LBW following tropical cyclone-related flooding (Fig. 8). Heterogeneity was reduced in this sub-analysis (I^2^ = 55%, *p*-value = 0.02).

Additional subgroup analyses were conducted to assess influence of geography and adjustment of covariates (Supplemental Figs. 1–2, Additional File 5). In a subgroup analysis of studies adjusting for maternal age and maternal education (N = 10), two of the most frequent included covariates (Table 3), heterogeneity is similar (I^2^ =70.0%, compared to I^2^= 77.3% for “good’ quality studies (Fig. 3) or I^2^ = 71.5% for all studies, Fig. 5 ), and the central effect size estimate is also similar, but no longer significant (RR = 1.05, 95% CI: 0.94, 1.18). When only U.S. studies (N = 11) were included in a subgroup analysis, I^2^ decreased to 64.0% and the estimated overall effect size was similar (RR = 1.01, 95% CI: 0.99, 1.03).

### Preterm birth

**Fig. 9:**
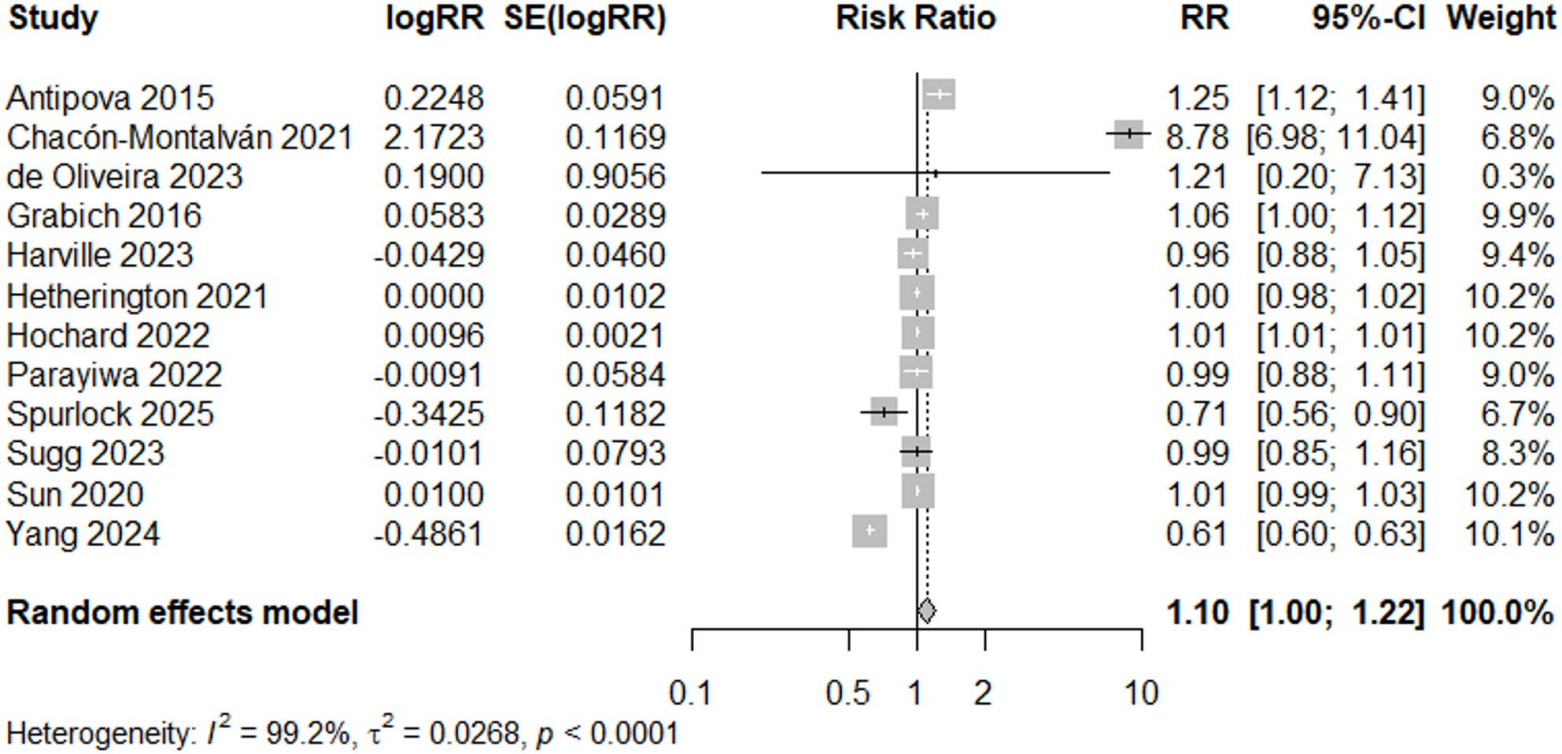
Forest Plot for PTB Studies, “Good” Quality Rating (*N* = 12)

**Fig. 10:**
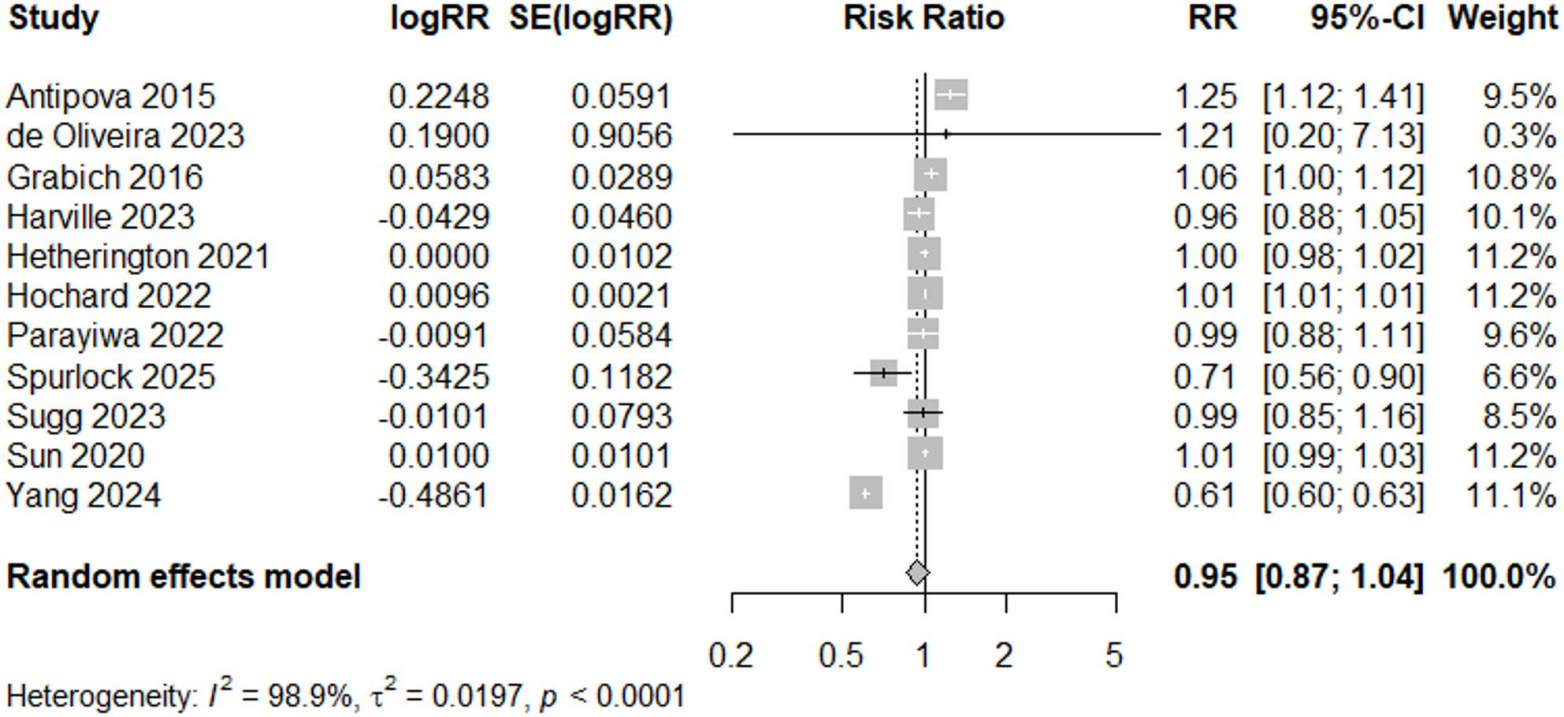
Forest Plot for PTB Studies, Sensitivity Analysis (*N* = 11)

Flooding was associated with an 10% increased risk of preterm birth (95% CI: 1.00, 1.22) using a random-effects model, when filtering by “Good” quality studies (*N* = 12) (Fig. 9). Visual inspection of the funnel plot showed a fairly symmetrical distribution of studies, indicating no strong evidence of publication bias (Fig. 4c). Although one study fell outside the funnel (Chacón-Montalván (2021)) [36], this did not strongly skew the overall symmetry (*p* = 0.86, Egger’s test for funnel plot asymmetry); however removing this study resulted in an overall RR of 0.95 (95% CI: 0.87–1.04), demonstrating sensitivity of the “Good” quality meta-analysis results to the inclusion of Chacón-Montalván (2021) [36] (Fig. 10).

**Fig. 11:**
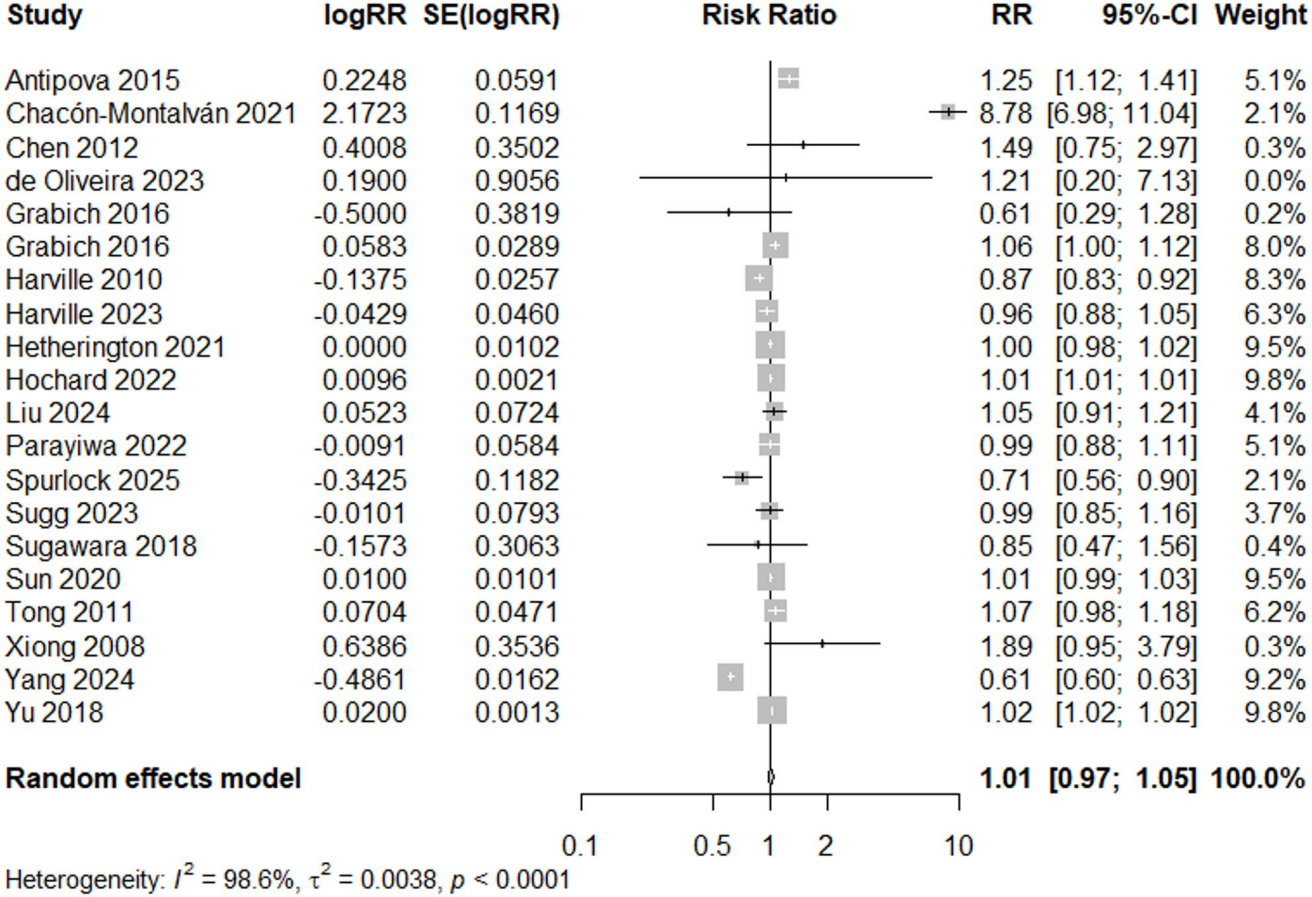
Forest Plot for PTB Studies, Overall (*N* = 20)

When studies with any quality rating are included (*N* = 20), the meta-analysis does not suggest a significant increase in PTB (RR = 1.01, 95% CI: 0.97, 1.05), and there is no indication of bias (*p* = 0.66, Egger’s test for funnel plot asymmetry) (Figs. 4d and 11). All results indicate high heterogeneity (I^2^ = 99%).

**Fig. 12:**
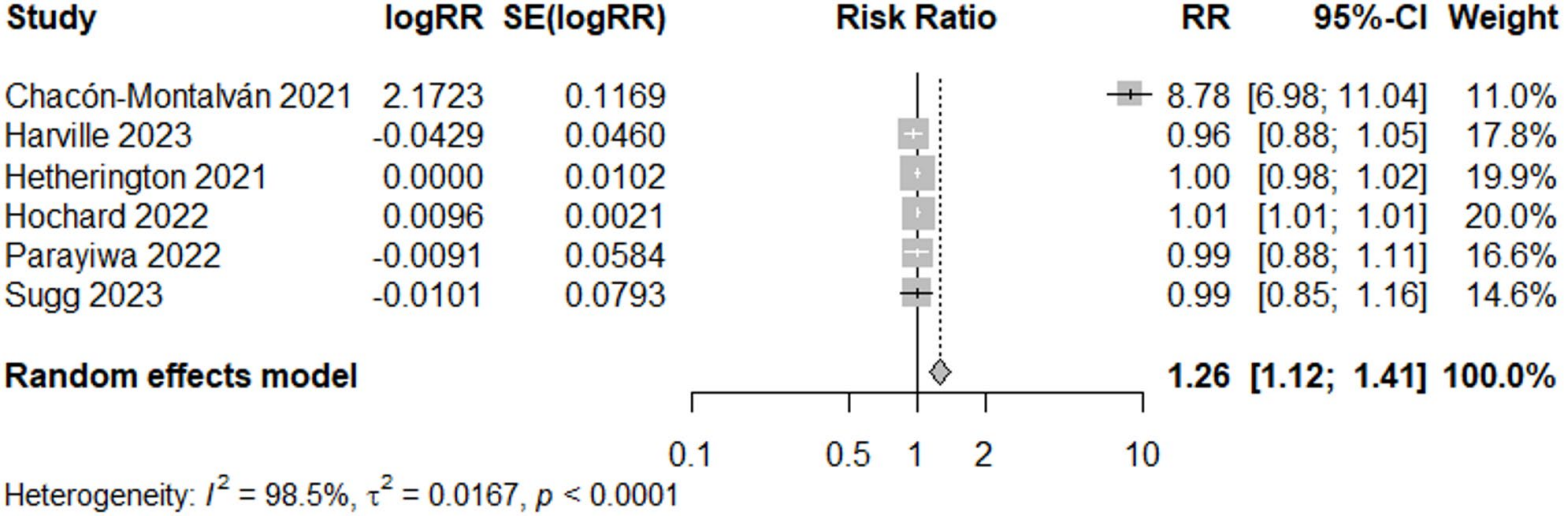
Forest Plot for PTB Studies, “Good” and Recent Event > 2005 (*N* = 6)

**Fig. 13:**
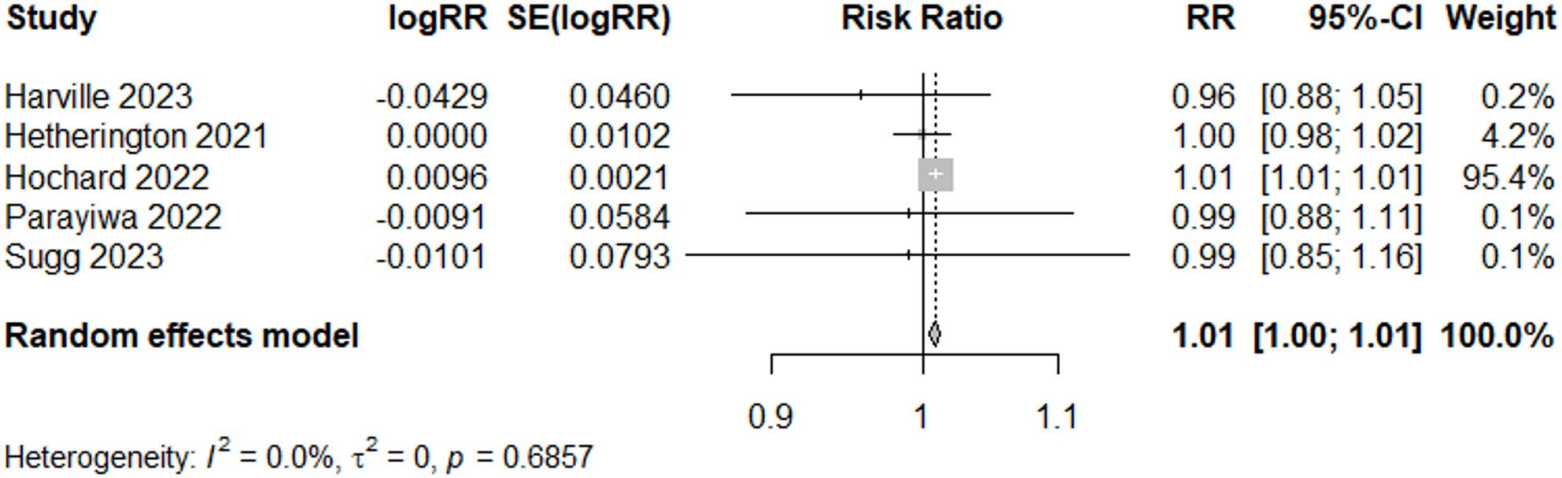
Forest Plot for PTB Studies, “Good” and Recent Event, Sensitivity Analysis removing Chacón-Montalván 2021 [36] (*N* = 5)

In the sub-analysis of good quality studies conducted for events after 2005 (*N* = 6), there was a 26% increased risk of preterm birth (RR = 1.26, 95% CI: 1.12, 1.41) (Fig. 12). Egger’s test for publication bias was not significant (*p* = 0.42). The estimated effect is influenced by Chacón-Montalván 2021 [36], as the estimated increase is small when this study is removed (RR = 1.01, 95% CI: 1.00, 1.01) (Fig. 13).

**Fig. 14:**
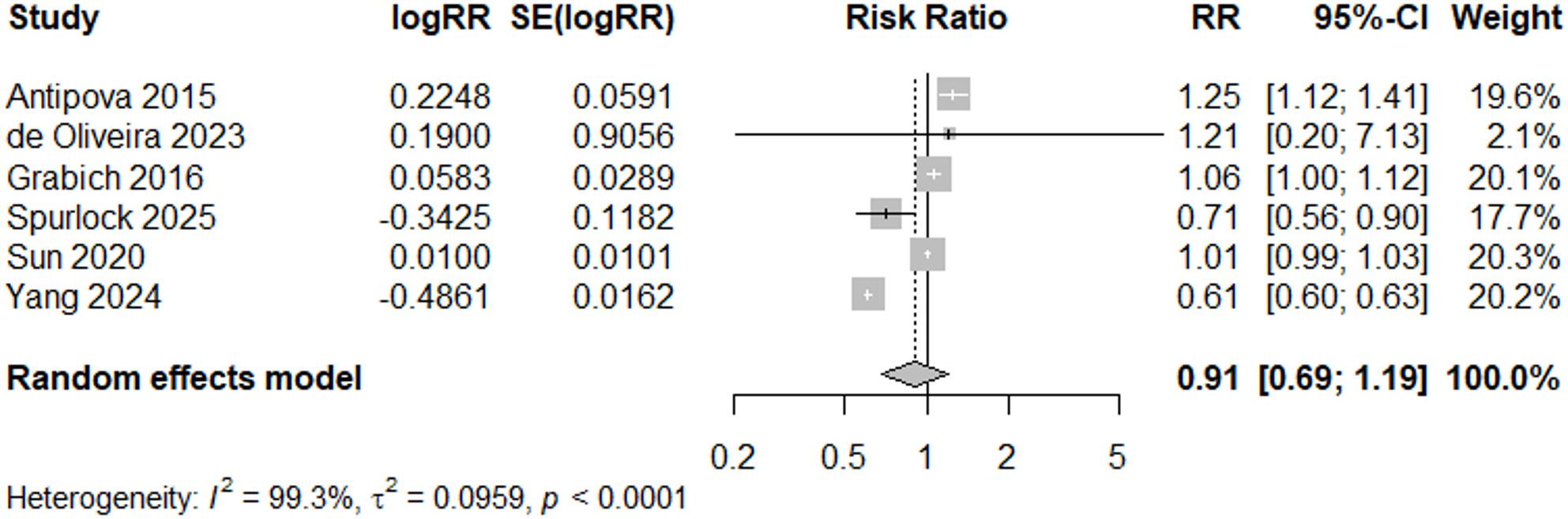
Forest Plot for PTB Studies, “Good” and Early Event ≤ 2005 (*N* = 6)

Among good quality studies with early event years (2005 or earlier) (*N* = 6), the overall estimated risk ratio was 0.91 (95% CI: 0.69, 1.19) (Fig. 14), suggesting no differences in estimates when dividing studies examining events before and following 2005. Heterogeneity was not present in meta-analysis of recent studies (I^2^ = 0%, *p* = 0.69), after removal of Chacón-Montalván 2021 [36], however heterogeneity remained high for the meta-analysis of studies of events during and before 2005 (I^2^ = 99%).

**Fig. 15:**
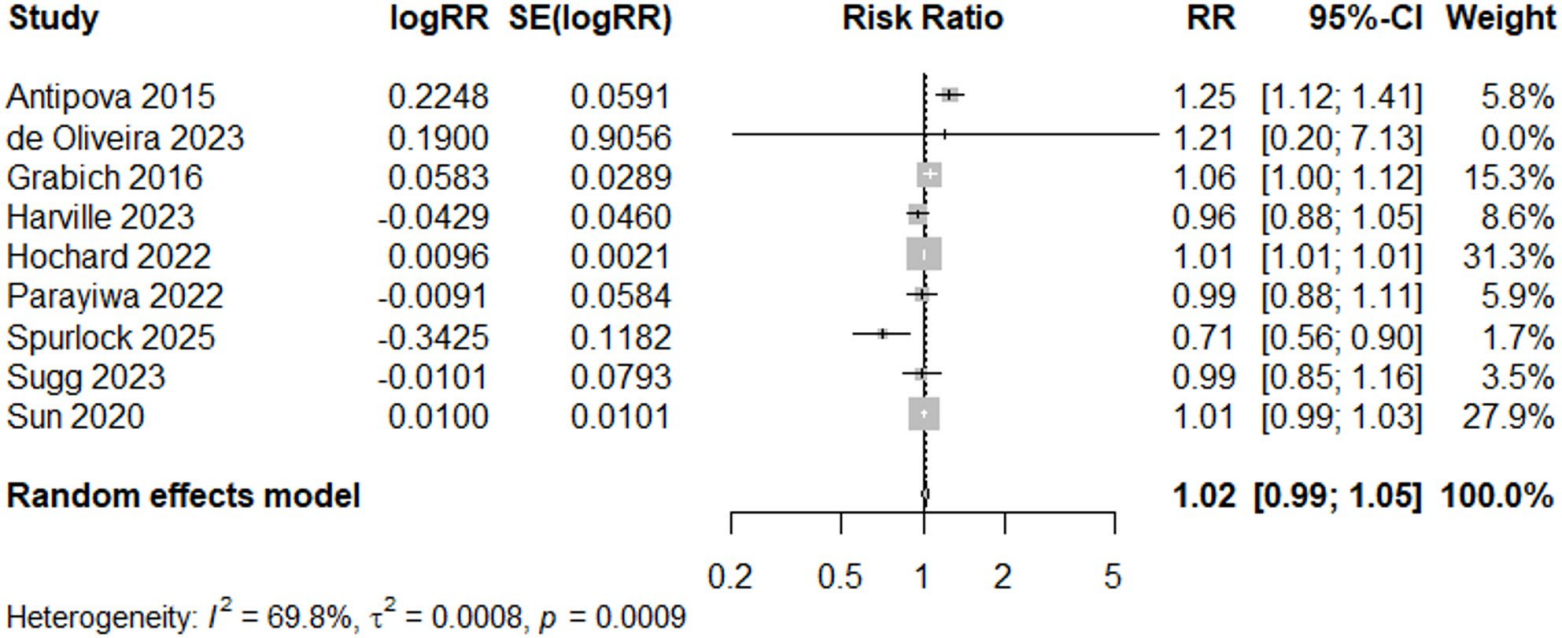
Forest Plot for PTB Studies, “Good” and TC-Related (*N* = 9)

A sub-analysis of good quality studies only including tropical cyclone-related flooding (*N* = 9) suggests a non-significant increase (RR = 1.02, 95% CI: 0.99, 1.05) in PTB following tropical cyclone-related flooding (Fig. 15). Heterogeneity was reduced in this sub-analysis (I^2^ = 70%), consistent with LBW findings, suggesting the type of event does explain some of the variation in effect sizes seen over all events.

As with LBW, additional subgroup analyses were conducted to assess influence of geography and adjustment of covariates (Supplemental Figs. 1–2, Additional File 5). In the subgroup analysis of studies adjusting for maternal age and maternal education (N = 9), heterogeneity is high (I^2^ =97.7%), similar to I^2^= 99.2% for “good’ quality studies (Fig. 9) or I^2^ = 98.6% for all studies (Fig. 11), and the central effect size estimate is higher (RR = 1.35, 95% CI: 0.98, 1.86) compared to “Good” Quality rating studies (Fig. 9) and all studies (Fig. 11). When only U.S. studies (N = 13) were included in a subgroup analysis, I^2^ decreased to 85.7% and the estimated overall effect size (RR = 1.01, 95% CI: 0.99, 1.02) was similar to the effect estimate for all studies (Fig. 11).

## Discussion

Overall, our present meta-analysis suggests a 3% (95% CI: 0–5%) increase in LBW and 10% (95% CI: 0–22%) increase in PTB following flood events, and the results for PTB are less consistent across studies. Funnel plots, particularly for PTB (Fig. 4c, d), indicate high variation in effect size estimates even among studies with relatively high sample size, with 5 of the 20 (and 4 of the 12 “good” quality rated studies) falling outside of the funnel. We expect this high heterogeneity is due, at least in part, to the inclusion of a wide range of flood exposure estimates, which is a limitation of the current analysis and reflects the wide range of methods used to estimate exposure to flooding in the current literature. In particular, Chacón-Montalván et al. (2021) [36] is the only study included that used rainfall intensity as the exposure metric and effect estimates are not consistent with other included studies. Chacón-Montalván et al. (2021) [36] also included a wider range of covariates in their models, which may also contribute to the difference in effect estimates. Removal of this study resulted in a smaller overall effect estimate for PTB (Fig. 13). Many studies examining tropical cyclone exposure focus on wind speed and/or disaster declarations to categorize exposure [33, 40–42, 44, 53]. Additionally, the spatial resolution for many of the exposure estimates is low and based on administrative boundaries (e.g. administrative level 2 (ADM2), corresponding to county in the United States) or ADM1 (corresponding to state in the United States) [37, 40–44, 46, 52–54]. This is often necessary given restrictions on accessing finer spatial resolution of home location from birth records or other health datasets, which is a common limitation in environmental epidemiology and likely contributes to heterogeneity in meta-analyses examining health outcomes associated with other environmental exposures [70, 71]. Future studies addressing the impact of exposure misclassification by estimating direct flood exposure at finer spatial resolution will aid in refining estimates of effect found in the present meta-analysis. Novel satellite-derived estimates may be particularly helpful in this regard [72, 73].

Sub-analyses did not suggest differences in effect based on type of flooding event and did not reduce heterogeneity substantially; however, a small sample size for non-tropical cyclone events hinders interpretation of these results. Future studies on inland and non-tropical cyclone-related events are warranted to further understand the specific effects of exposure to flooding with different types of warning systems in place (e.g. flash flooding versus tropical cyclone-related flooding). The stronger association between flood exposure and low birthweight in studies after 2005 may reflect improved exposure estimation or higher risk events, suggesting further investigation is warranted to determine contributing factors.

This systematic review and meta-analysis revealed limited research elucidating social or biological mechanisms mediating the relationship between LBW and/or PTB and *in utero* exposure to flooding events. While premature initiation of labor by stress is a potential common mechanism linking extreme weather events to PTB generally [8], there may be specific infectious or chemical exposures during flood events that may also increase the risk of PTB [74, 75]. Additional factors that may play a role and could be the focus of future mediation analyses include disruption of prenatal care, maternal infections, maternal psychological stress, and regional healthcare resources. For LBW not coincident with PTB (term LBW), disruption of food resources leading to reduced fetal growth has been suggested as a mediator between flood events and LBW, particularly in low resource settings [9, 67, 68].

Our results are limited by the small number of studies using similar methods for exposure estimation, potential exposure misclassification due to the low spatial resolution at which most flood exposure estimates were available, and potential co-exposures (e.g. wind damage during tropical cyclones). Future studies could focus on examining term LBW and/or small for gestational age (SGA), in addition to PTB and LBW outcomes, to determine the relative importance of acute stress leading to initiation of preterm labor versus long-term impacts on fetal growth. Acceptance and more widespread application of these standardized dichotomous outcomes, in addition to PTB and LBW, would support future meta-analyses, as eight studies in the present analysis had to be excluded simply because the dichotomous outcomes of PTB or LBW were not calculated from the available birthweight and gestational age datasets (Table 1).

## Conclusion

Results of this systematic review and meta-analysis indicate that LBW and PTB may increase following *in utero* exposure to flood events. This meta-analysis provides a summary of a growing literature examining impacts of flooding on PTB and LBW and identifies important methodological considerations for future epidemiological investigations. Future studies incorporating finer spatiotemporally resolved estimates of exposure to flooding and potential mediators between flooding and birth outcomes will improve our understanding of birth outcomes following exposure to flooding during pregnancy.

## Supplementary Information

Additional file 1: Search Strategy. Description of data: Search strategy details including search dates, databases, terms and yields.

Additional file 2: Supplementary Tables. Description of data: Tables detailing study characteristics, meta-analysis results, full data extraction and transformations, and quality assessment.

Additional file 3: Cohen’s Kappa scores for Interrater reliability screening steps.

Additional file 4: R Code. Description of data: R code used for meta-analyses.

Additional file 5: Subgroup forest plots for covariates and U.S. only studies.

## Data Availability

The datasets supporting the conclusions of this article are included within the article and its additional files.

## Abbreviations

A: Adjusted
ACR: Assumed comparator risk
ADM: Administrative level
BMI: Body Mass Index
BW: Birthweight
CI: Confidence interval
DDD: Difference in difference in difference
DID: Difference in difference
F: Flood, inland, riverine
FEMA: Federal Emergency Management Agency
GA: Gestational age
GBD: Global burden of disease
HR: Hazard ratio
I^2^: Percentage of the variability in meta-analysis effect estimate that is due to heterogeneity rather than sampling error
IRR: Inter-rater reliability
LBW: Low birthweight
MECCIR: Campbell Collaboration’s Methodological Expectations
NR: Not reported
O: Other (i.e. monsoon, tsunami, direct measure of precipitation)
OR: Odds ratio
PRISMA: Preferred Reporting Items for Systematic Reviews and Meta-Analysis
PTB: Preterm birth
Qld: Queensland
RD: Risk difference
RR: Risk ratio
RRR: Relative risk reduction
SE: Standard error
TC: Tropical cyclone, hurricane, typhoon

## References

[1] Tunstall S, Tapsell S, Green C, Floyd P, George C. The health effects of flooding: social research results from England and Wales. J Water Health. 2006;4(3):365–80. 17036844 10.2166/wh.2006.031

[2] Fernandez A, Black J, Jones M, Wilson L, Salvador-Carulla L, Astell-Burt T, et al. Flooding and mental health: a systematic mapping review. PLoS ONE. 2015;10(4):e0119929. 25860572 10.1371/journal.pone.0119929 PMC4393088

[3] Du W, FitzGerald GJ, Clark M, Hou XY. Health impacts of floods. Prehosp Disaster Med. 2012;25(3):265–72. 10.1017/s1049023x00008141 20586021

[4] Erickson TB, Brooks J, Nilles EJ, Pham PN, Vinck P. Environmental health effects attributed to toxic and infectious agents following hurricanes, cyclones, flash floods and major hydrometeorological events. J Toxicol Environ Health B Crit Rev. 2019;22(5–6):157–71. 31437111 10.1080/10937404.2019.1654422

[5] Nour NN. Maternal health considerations during disaster relief. Rev Obstet Gynecol. 2011;4(1):22–7. 21629495 PMC3100103

[6] Lowe D, Ebi KL, Forsberg B. Factors increasing vulnerability to health effects before, during and after floods. Int J Environ Res Public Health. 2013;10(12):7015–67. 24336027 10.3390/ijerph10127015 PMC3881153

[7] Tate E, Rahman MA, Emrich CT, Sampson CC. Flood exposure and social vulnerability in the united States. Nat Hazards. 2021;106(1):435–57.

[8] Sandman CA, Davis EP, Buss C, Glynn LM. Exposure to prenatal Psychobiological stress exerts programming influences on the mother and her fetus. Neuroendocrinology. 2012;95(1):8–21. 10.1159/000327017 PMC7068789 21494029

[9] Mallett PhD LH, Etzel MD, RA PhD. Flooding: what is the impact on pregnancy and child health? Disasters. 2018;42(3):432–58. 29057549 10.1111/disa.12256

[10] Harville EW, Xiong X, Pridjian G, Elkind-Hirsch K, Buekens P. Postpartum mental health after hurricane katrina: a cohort study. BMC Pregnancy Childbirth. 2009;9(1):21. 19505322 10.1186/1471-2393-9-21 PMC2702337

[11] Zahran S, Magzamen S, Breunig IM, Mielke HW. Maternal exposure to neighborhood soil Pb and eclampsia risk in new Orleans, Louisiana (USA): evidence from a natural experiment in flooding. Environ Res. 2014;133:274–81. 24981826 10.1016/j.envres.2014.06.007

[12] Hirabayashi Y, Mahendran R, Koirala S, Konoshima L, Yamazaki D, Watanabe S, et al. Global flood risk under climate change. Nat Clim Chang. 2013;3(9):816–21.

[13] Saulnier DD, Brolin K. A systematic review of the health effects of prenatal exposure to disaster. Int J Public Health. 2015;60:781–7. 26298438 10.1007/s00038-015-0699-2

[14] Huang W, Gao Y, Xu R, Yang Z, Yu P, Ye T et al. Health effects of cyclones: a systematic review and meta-analysis of epidemiological studies. Environ Health Perspect. 2023;131(8):086001. 10.1289/EHP12158 PMC10461789 37639476

[15] Zotti ME, Williams AM, Robertson M, Horney J, Hsia J. Post-disaster reproductive health outcomes. Matern Child Health J. 2013;17:783–96. 22752348 10.1007/s10995-012-1068-x PMC4540175

[16] Segal TR, Giudice LC. Systematic review of climate change effects on reproductive health. Fertil Steril. 2022;118(2):215–23. 35878942 10.1016/j.fertnstert.2022.06.005

[17] Jeffers NK, Glass N. Integrative review of pregnancy and birth outcomes after exposure to a hurricane. J Obstet Gynecol Neonatal Nurs. 2020;49(4):348–60. 32553921 10.1016/j.jogn.2020.04.006

[18] Partash N, Naghipour B, Rahmani SH, Asl YP, Arjmand A, Ashegvatan A, et al. The impact of flood on pregnancy outcomes: a review Article. Taiwan J Obstet Gynecol. 2022;61(1):10–4. 35181015 10.1016/j.tjog.2021.11.005

[19] Harville EW, Beitsch L, Uejio CK, Sherchan S, Lichtveld MY. Assessing the effects of disasters and their aftermath on pregnancy and infant outcomes: a conceptual model. Int J Disaster Risk Reduct. 2021;62:102415. 10.1016/j.ijdrr.2021.102415 PMC8318346 34336567

[20] Aloe AM, Dewidar O, Hennessy EA, Pigott T, Stewart G, Welch V et al. Campbell standards: modernizing Campbell’s methodologic expectations for Campbell Collaboration intervention reviews (MECCIR). Campbell Syst Rev. 202;20(4):e1445. 10.1002/cl2.1445 PMC11456310 39376895

[21] PROSPERO. Available from: https://www.crd.york.ac.uk/PROSPERO/view/CRD42024514540.

[22] Page MJ, Moher D, Bossuyt PM, Boutron I, Hoffmann TC, Mulrow CD, et al. PRISMA 2020 explanation and elaboration: updated guidance and exemplars for reporting systematic reviews. BMJ. 2021;372:n160. 33781993 10.1136/bmj.n160 PMC8005925

[23] World Health Organization. Preterm birth. Available from: https://www.who.int/news-room/fact-sheets/detail/preterm-birth.

[24] World Health Organization. Low birth weight. Available from: https://www.who.int/data/nutrition/nlis/info/low-birth-weight.

[25] PRISMA for searching. PRISMA Statement. Available from: https://www.prisma-statement.org/prisma-search.

[26] Haddaway NR, Grainger MJ, Gray CT. citationchaser: An R package and Shiny app for forward and backward citations chasing in academic searching. Zenodo; 2021. Available from: https://zenodo.org/records/4543513.

[27] Veritas Health Innovation. Covidence systematic review software. Covidence. Available from: https://www.covidence.org.

[28] Study quality assessment tools. NIH National Heart Lung, and Blood Institute. Available from: https://www.nhlbi.nih.gov/health-topics/study-quality-assessment-tools.

[29] MetaQAT – critical appraisal tool. Public Health Ontario. Available from: http://www.publichealthontario.ca/en/Health-Topics/Public-Health-Practice/Library-Services/MetaQAT.

[30] Higgins J, Thomas J, Chandler J, Cumpston M, Li T, Page M et al. Cochrane handbook for systematic reviews of interventions version 6.5. Cochrane; 2024. Available from: https://training.cochrane.org/handbook. 10.1002/14651858.ED000142 PMC10284251 31643080

[31] Schwarzer G, Carpenter JR, Rücker G. Meta-analysis with R. Cham: Springer International Publishing; 2015. (Use R!). Available from: https://link.springer.com//10.1007/978-3-319-21416-0.

[32] Egger M, Smith GD, Schneider M, Minder C. Bias in meta-analysis detected by a simple, graphical test. BMJ. 1997; Available from: https://www.bmj.com/content/315/7109/629. 10.1136/bmj.315.7109.629 PMC2127453 9310563

[33] Antipova A, Curtis A. The post-disaster negative health legacy: pregnancy outcomes in Louisiana after hurricane Andrew. Disasters. 2015;39(4):665–86. 25754615 10.1111/disa.12125

[34] Beuermann DW, Pecha C, Schmid JP. The effects of weather shocks on early childhood development. Washington, DC: Inter-American Development Bank, Country Department Caribbean Group; 2017.

[35] Biswas S, Mondal S, Banerjee A, Alam A, Satpati L. Investigating the association between floods and low birth weight in india: using the Geospatial approach. Sci Total Environ. 2024;912:169593. 38151131 10.1016/j.scitotenv.2023.169593

[36] Chacón-Montalván EA, Taylor BM, Cunha MG, Davies G, Orellana JDY, Parry L. Rainfall variability and adverse birth outcomes in Amazonia. Nat Sustain. 2021;4:583–94.

[37] Chen CK, Matthews-Juarez P, Yang A. Effect of hurricane katrina on low birth weight and preterm deliveries in African American women in Louisiana, Mississippi, and Alabama. J Syst Cybern Inf. 2012;10(2):102–7.

[38] Currie J. Weathering the storm: hurricanes and birth outcomes. J Health Econ. 2013;32(3):487–503. 23500506 10.1016/j.jhealeco.2013.01.004 PMC3649867

[39] de Oliveira VH, Lee I, Quintana-Domeque C. Natural disasters and early human development: hurricane Catarina and infant health in Brazil. J Hum Resour. 2023;58(3):819–51.

[40] Grabich SC, Horney J, Konrad CE, Lobdell D. Measuring the storm: methods of quantifying hurricane exposure with pregnancy outcomes. Nat Hazards Rev. 2016;17(1):06015002.

[41] Grabich SC, Robinson WR, Engel SM, Konrad CE, Richardson DB, Horney JA. Hurricane Charley exposure and hazard of preterm delivery, Florida 2004. Matern Child Health J. 2016;20(12):2474–82. 27485492 10.1007/s10995-016-2069-y

[42] Grabich SC, Robinson WR, Konrad CE, Horney JA. Impact of hurricane exposure on reproductive health outcomes, Florida, 2004. Disaster Med Public Health Prep. 2017;11(4):407–11. 28093094 10.1017/dmp.2016.158

[43] Harville EW, Tran T, Xiong X, Buekens P. Population changes, racial/ethnic disparities, and birth outcomes in Louisiana after hurricane katrina. Disaster Med Public Health Prep. 2010;4(Suppl 1):S39–45. 23105034 10.1001/dmp.2010.15

[44] Harville EW, Pan K, Beitsch L, Sherchan SP, Gonsoroski E, Uejio C, et al. Hurricane Michael and adverse birth outcomes in the Florida panhandle: analysis of vital statistics data. Disaster Med Public Health Prep. 2023;17(e94):1–8. 10.1017/dmp.2021.367 PMC9440161 35236537

[45] Hetherington E, Adhikari K, Tomfohr-Madsen L, Patten S, Metcalfe A. Birth outcomes, pregnancy complications, and postpartum mental health after the 2013 Calgary flood: a difference in difference analysis. PLoS ONE. 2021;16(2):e0246670. 33571314 10.1371/journal.pone.0246670 PMC7877569

[46] Hochard J, Li Y, Abashidze N. Associations of hurricane exposure and forecasting with impaired birth outcomes. Nat Commun. 2022;13(1):6746. 10.1038/s41467-022-33865-x PMC9643368 36347839

[47] Liu X, Berberian AG, Wang S, Cushing LJ. Hurricane Harvey and the risk of spontaneous preterm and early-term birth. Environ Epidemiol. 2024;8(3):e312. 10.1097/EE9.0000000000000312 PMC11115986 38799265

[48] Nasir M. Prenatal exposure to shocks and early-life health: impact of terrorism and flood on birth outcomes in Pakistan, Islamabad. Pakistan: Pakistan Institute of Development Economics; 2018 p. 572–87. Available from: https://file.pide.org.pk/pdf/Working%20Paper/WorkingPaper-155.pdf.

[49] Parayiwa C, Harley D, Clark R, Behie A, Lal A. Association between severe cyclone events and birth outcomes in Queensland, Australia, 2008–2018: a population based retrospective cohort study. Aust N Z J Public Health. 2022;46(6):835–41. 35735907 10.1111/1753-6405.13273

[50] Spurlock T, Guignet D, Runkle JD, Sugg MM. Examining hurricane exposure on neonatal outcomes in North Carolina: a case study of hurricane Isabel in 2003. Int J Disaster Risk Reduct. 2025;116:105075.

[51] Sugawara J, Iwama N, Hoshiai T, Tokunaga H, Nishigori H, Metoki H, et al. Regional birth outcomes after the 2011 great East Japan earthquake and tsunami in Miyagi Prefecture. Prehosp Disaster Med. 2018;33(2):215–9. 29560850 10.1017/S1049023X18000183

[52] Sugg MM, Runkle JD, Ryan SC, Wertise L. A difference-in difference analysis of the South Carolina 2015 extreme floods and the association with maternal health. Int J Disaster Risk Reduct. 2023;97:104037. 10.1016/j.ijdrr.2023.104037 PMC10956501 38525445

[53] Sun S, Weinberger KR, Yan M, Anderson GB. Tropical cyclones and risk of preterm birth: a retrospective analysis of 20 million births across 378 US counties. Environ Int. 2020;140:105825. 10.1016/j.envint.2020.105825 32485474

[54] Tong VT, Zotti ME, Hsia J. Impact of the red river catastrophic flood on women giving birth in North Dakota, 1994–2000. Matern Child Health J. 2011;15(3):281–8. 20204482 10.1007/s10995-010-0576-9

[55] Xiong X, Harville EW, Mattison DR, Elkind-Hirsch K, Pridjian G, Buekens P. Exposure to hurricane Katrina, post-traumatic stress disorder and birth outcomes. Am J Med Sci. 2008;336(2):111–5. 18703903 10.1097/MAJ.0b013e318180f21c PMC2635112

[56] Yang Z, Huang W, McKenzie J, Yu P, Wu Y, Xu R, et al. The association of adverse birth outcomes with flood exposure before and during pregnancy in australia: a cohort study. Lancet Planet Health. 2024;8(8):e554–63. 39122324 10.1016/S2542-5196(24)00142-6

[57] Yu X, Feric Z, Cordero J, Meeker J, Alshawabkeh A. Potential influence of temperature and precipitation on preterm birth rate in Puerto Rico. Sci Rep. 2018;8(1):16106. 10.1038/s41598-018-34179-z PMC6208375 30382121

[58] Baloch S, Khaskheli MN, Sheeba A. Screening of reproductive health problems in flood affected pregnant women. J Liaquat Univ Med Health Sci. 2012;11(2):101–4.

[59] Hamilton BE, Sutton PD, Mathews TJ, Martin JA, Ventura SJ. The effect of hurricane katrina: births in the U.S. Gulf Coast region, before and after the storm. Natl Vital Stat Rep. 2009;58(2):1–28. 19754006

[60] Hilmert CJ, Kvasnicka-Gates L, Teoh AN, Bresin K, Fiebiger S. Major flood related strains and pregnancy outcomes. Health Psychol. 2016;35(11):1189. 10.1037/hea0000386 27280371

[61] Ishikuro M, Noda A, Murakami K, Onuma T, Matsuzaki F, Ueno F, et al. Families’ health after the great East Japan earthquake: findings from the Tohoku medical megabank project birth and Three-Generation cohort study. J Epidemiol. 2022;256(2):93–10. 10.1620/tjem.256.93 35197407

[62] Kroska EB, O’Hara MW, Elgbeili G, Hart KJ, Laplante DP, Dancause KN et al. The impact of maternal flood-related stress and social support on offspring weight in early childhood. Arch Womens Ment Health. 2018;21:225–33. 10.1007/s00737-017-0786-x PMC7318548 29080991

[63] Le K, Nguyen M. The impacts of rainfall shocks on birth weight in Vietnam. J Dev Effect. 2022;14(2):143–59.

[64] Morrow S. Typhoons and lower birth weight in the Philippines [Master’s Theses]. The University of San Francisco; 2014. Available from: https://repository.usfca.edu/thes/89.

[65] Nguyen M, Le K. Rainfall and birth outcome: evidence from Kyrgyzstan. Clim Dev. 2023;15(1):20–9.

[66] Sanguanklin N, McFarlin BL, Park CJ, Giurgescu C, Finnegan L, White-Traut R, et al. Effects of the 2011 flood in Thailand on birth outcomes and perceived social support. J Obstet Gynecol Neonatal Nurs. 2014;43(4):435–44. 24956975 10.1111/1552-6909.12466

[67] Pappas A, Kovats S, Ranganathan M. Extreme weather events and maternal health in low-income and middle-income countries: a scoping review. BMJ Open. 2013;14(6):e079361. 10.1136/bmjopen-2023-079361 PMC11149126 38830734

[68] Safajou F, Nahidi F, Ahmadi F. Reproductive health challenges during a flood: a qualitative study. Nurs Open. 2024;11(1):e2044. 38268287 10.1002/nop2.2044 PMC10697115

[69] Nick GA, Savoia E, Elqura L, Crowther MS, Cohen B, Leary M, et al. Emergency preparedness for vulnerable populations: people with special health-care needs. Public Health Rep. 2009;124(2):338–43. 19320378 10.1177/003335490912400225 PMC2646456

[70] Kondo MC, De Roos AJ, White LS, Heilman WE, Mockrin MH, Gross-Davis CA, et al. Meta-analysis of heterogeneity in the effects of wildfire smoke exposure on respiratory health in North America. Int J Environ Res Public Health. 2019;16(6):960. 30889810 10.3390/ijerph16060960 PMC6466235

[71] Guo P, Warren JL, Deziel NC, Liew Z. Exposure range matters: considering nonlinear associations in the meta-analysis of environmental pollutant exposure using examples of per-and polyfluoroalkyl substances and birth outcomes. Am J Epidemiol. 2025;194(4):1043–51. 39227151 10.1093/aje/kwae309

[72] Tellman B, Sullivan JA, Kuhn C, Kettner AJ, Doyle CS, Brakenridge GR, et al. Satellite imaging reveals increased proportion of population exposed to floods. Nature. 2021;596(7870):80–6. 34349288 10.1038/s41586-021-03695-w

[73] Composto RW, Tulbure MG, Tiwari V. Quantifying urban flood extent using satellite imagery and machine learning. Nat Hazards. 2025;121:171–99.

[74] Cruz AM, Krausmann E. Hazardous materials releases from offshore oil and gas facilities and emergency response following hurricanes katrina and Rita. J Loss Prev Process Ind. 2009;22(1):59–65.

[75] Cutts BB, Vilá O, Bray LA, Harris A, Hornsby G, Goins H et al. Shifting terrains: Understanding residential contaminants after flood disasters. Sci Total Environ. 2024;907:167577. 10.1016/j.scitotenv.2023.167577 37839486

