# Supplementary material for: The effect of flooding on low birthweight and preterm birth: a systematic review and meta-analysis
Source: BMC Public Health. 2026 Mar 5;26:800. doi: 10.1186/s12889-026-26521-2 (PMC12961868; doi:10.1186/s12889-026-26521-2)
Supplement: Supplementary file 1 — Additional file 1: Search Strategy. Description of data: Search strategy details including search dates, databases, terms and yields. [file 12889_2026_26521_MOESM1_ESM.docx]

### Summary of Results

All initial searches were implemented on April 4, 2024.

All search updates were implemented on February 23, 2025.

No search filters were applied in any database.

| Platform | Database | Date of Coverage | Total Yield (Initial) | Total Yield (Update) | Unique Yield from Update |
| --- | --- | --- | --- | --- | --- |
| CABI | CAB Abstracts | 1910s-present | 792 | 826 | 47 |
| EBSCOhost | Academic Search Complete | 1980s-present | 844 | 885 | 56 |
|  | Environment Complete | 1888-present | 178 | 189 | 17 |
| ProQuest | Environmental Science Index & Environmental Science Database* | 1975-present | 572 | 590 | 33 |
| PubMed | | 1940-present | 726 | 769 | 63 |
| Scopus | | 1800s-present | 1,688 | 1,792 | 167 |
| Web of Science | Web of Science Core Collection | 1900-present | 1,098 | 1,199 | 167 |
| Total yield (initial search) | | | 5,898 | - | - |
| Deduplicated yield (initial search) | | | 3,195 | - | - |
| Total yield (search update) | | | - | - | 550 |
| Deduplicated yield (search update) | | | - | - | 364 |

*Multiple databases searched, including: Bacteriology Abstracts (Microbiology B), Biotechnology Research Abstracts, Biotechnology Research Abstracts, Ecology Abstracts, Environmental Engineering Abstracts, Environmental Science Collection, Health & Safety Science Abstracts, Industrial and Applied Microbiology Abstracts (Microbiology A), Toxicology Abstracts, TOXLINE

### Academic Search Complete (EBSCOhost)

| 1 | TI ( (Hurricane* OR Cyclone* OR Flood* OR Storm* OR Rainstorm* OR Thunderstorm* OR Superstorm* OR Tempest* OR “Heavy Rain*” OR Monsoon* OR Typhoon* OR “Tidal wave*” OR tidalwave* OR inundation* OR tsunami* OR meteotsunami* OR deluge*) ) OR AB ( (Hurricane* OR Cyclone* OR Flood* OR Storm* OR Rainstorm* OR Thunderstorm* OR Superstorm* OR Tempest* OR “Heavy Rain*” OR Monsoon* OR Typhoon* OR “Tidal wave*” OR tidalwave* OR inundation* OR tsunami* OR meteotsunami* OR deluge*) ) OR KW ( (Hurricane* OR Cyclone* OR Flood* OR Storm* OR Rainstorm* OR Thunderstorm* OR Superstorm* OR Tempest* OR “Heavy Rain*” OR Monsoon* OR Typhoon* OR “Tidal wave*” OR tidalwave* OR inundation* OR tsunami* OR meteotsunami* OR deluge*) ) | 280,063 |
| --- | --- | --- |
| 2 | (DE "CYCLONES" OR DE "CYCLONE Aila, 2009" OR DE "CYCLONE Bola, 1988" OR DE "CYCLONE Colina, 1993" OR DE "CYCLONE Gonu, 2007" OR DE "CYCLONE Heta, 2004" OR DE "CYCLONE Nadia, 1994" OR DE "CYCLONE Nargis, 2008" OR DE "CYCLONE Orissa, 1999" OR DE "CYCLONE Sidr, 2007" OR DE "CYCLONE Tracy, 1974" OR DE "CYCLONE Winifred, 1986" OR DE "CYCLONE Yasi, 2011" OR DE "ERWIN Storm, 2005" OR DE "KLAUS Storm, 2009" OR DE "LOTHAR Storm, 1999" OR DE "TORNADOES" OR DE "TROPICAL cyclones" OR DE "TYPHOONS" OR DE "STORMS" OR DE "CYCLONES" OR DE "RAINSTORMS" OR DE "THUNDERSTORMS" OR DE "HURRICANES" OR DE "GALVESTON (Tex.) hurricane, 1900" OR DE "HURRICANE Agnes, 1972" OR DE "HURRICANE Alicia, 1983" OR DE "HURRICANE Andrew, 1992" OR DE "HURRICANE Audrey, 1957" OR DE "HURRICANE Belle, 1976" OR DE "HURRICANE Bertha, 1996" OR DE "HURRICANE Betsy, 1965" OR DE "HURRICANE Beulah, 1967" OR DE "HURRICANE Bob, 1991" OR DE "HURRICANE Bonnie, 1998" OR DE "HURRICANE Camille, 1969" OR DE "HURRICANE Carla, 1961" OR DE "HURRICANE Carol, 1954" OR DE "HURRICANE Charley, 2004" OR DE "HURRICANE David, 1979" OR DE "HURRICANE Dean, 2007" OR DE "HURRICANE Debby, 1982" OR DE "HURRICANE Dennis, 1999" OR DE "HURRICANE Dennis, 2005" OR DE "HURRICANE Diana, 1984" OR DE "HURRICANE Diane, 1955" OR DE "HURRICANE Dolly, 2008" OR DE "HURRICANE Donna, 1960" OR DE "HURRICANE Dora, 1964" OR DE "HURRICANE Dorian, 2019" OR DE "HURRICANE Earl, 1998" OR DE "HURRICANE Edna, 1954" OR DE "HURRICANE Elena, 1985" OR DE "HURRICANE Emily, 1954" OR DE "HURRICANE Emily, 1993" OR DE "HURRICANE Emily, 2005" OR DE "HURRICANE Erin, 1995" OR DE "HURRICANE Felix, 1995" OR DE "HURRICANE Felix, 2007" OR DE "HURRICANE Flora, 1963" OR DE "HURRICANE Florence, 1988" OR DE "HURRICANE Florence, 2018" OR DE "HURRICANE Floyd, 1999" OR DE "HURRICANE Fran, 1996" OR DE "HURRICANE Frances, 2004" OR DE "HURRICANE Frederic, 1979" OR DE "HURRICANE Georges, 1998" OR DE "HURRICANE Gilbert, 1988" OR DE "HURRICANE Ginger, 1971" OR DE "HURRICANE Gloria, 1985" OR DE "HURRICANE Gordon, 1994" OR DE "HURRICANE Gustav, 2008" OR DE "HURRICANE Harvey, 2017" OR DE "HURRICANE Hattie, 1961" OR DE "HURRICANE Hazel, 1954" OR DE "HURRICANE Hilda, 1955" OR DE "HURRICANE Hugo, 1989" OR DE "HURRICANE Idalia, 2023" OR DE "HURRICANE Igor, 2010" OR DE "HURRICANE Ike, 2008" OR DE "HURRICANE Iniki, 1992" OR DE "HURRICANE Irene, 1999" OR DE "HURRICANE Irene, 2011" OR DE "HURRICANE Iris, 2001" OR DE "HURRICANE Irma, 2017" OR DE "HURRICANE Isaac, 2012" OR DE "HURRICANE Isabel, 2003" OR DE "HURRICANE Isidore, 2002" OR DE "HURRICANE Ivan, 2004" OR DE "HURRICANE Iwa, 1982" OR DE "HURRICANE Janet, 1955" OR DE "HURRICANE Jeanne, 2004" OR DE "HURRICANE Joan, 1988" OR DE "HURRICANE Juan, 1985" OR DE "HURRICANE Juan, 2003" OR DE "HURRICANE Kate, 1985" OR DE "HURRICANE Katrina, 2005" OR DE "HURRICANE Lenny, 1999" OR DE "HURRICANE Luis, 1995" OR DE "HURRICANE Maria, 2017" OR DE "HURRICANE Marilyn, 1995" OR DE "HURRICANE Matthew, 2016" OR DE "HURRICANE Michael, 2018" OR DE "HURRICANE Mitch, 1998" OR DE "HURRICANE Norbert, 1984" OR DE "HURRICANE Opal, 1995" OR DE "HURRICANE Patricia, 2015" OR DE "HURRICANE Pauline, 1997" OR DE "HURRICANE Rita, 2005" OR DE "HURRICANE Sandy, 2012" OR DE "HURRICANE Stan, 2005" OR DE "HURRICANE Wilma, 2005" OR DE "HURRICANE damage" OR DE "HURRICANE modification" OR DE "HURRICANE protection" OR DE "HURRICANE tracks" OR DE "HURRICANES & architecture" OR DE "LABOR Day Hurricane, 1935" OR DE "NEW England Hurricane, 1938" OR DE "OKEECHOBEE Hurricane, 1928" OR DE "MONSOONS" OR DE "MALAYSIAN-Australian monsoons" OR DE "NORTH American Monsoons" OR DE "WEST African monsoons" OR DE "FLOODS" OR DE "TYPHOONS" OR DE "SUPER Typhoon Haiyan, 2013" OR DE "TYPHOON Mangkhut, 2018" OR DE "TYPHOON Ondoy, 2009" OR DE "TYPHOON Sinlaku, 2002" OR DE "TSUNAMIS" OR DE "HUNGA Tonga-Hunga Ha'apai Eruption & Tsunami, 2022" OR DE "INDIAN Ocean Tsunami, 2004" OR DE "METEOTSUNAMIS") | 82,771 |
| 3 | TI ( (Preterm* OR Birth* OR Lowbirth* OR Gestation* OR ((Pregnan* OR Prenatal* OR pre-natal* OR Perinatal* OR peri-natal* OR “in utero*”) N15 (Time OR Timing OR Duration OR Length OR Span OR interval* OR period* OR weight*))) ) OR AB ( (Preterm* OR Birth* OR Lowbirth* OR Gestation* OR ((Pregnan* OR Prenatal* OR pre-natal* OR Perinatal* OR peri-natal* OR “in utero*”) N15 (Time OR Timing OR Duration OR Length OR Span OR interval* OR period* OR weight*))) ) OR KW ( (Preterm* OR Birth* OR Lowbirth* OR Gestation* OR ((Pregnan* OR Prenatal* OR pre-natal* OR Perinatal* OR peri-natal* OR “in utero*”) N15 (Time OR Timing OR Duration OR Length OR Span OR interval* OR period* OR weight*))) ) | 421,723 |
| 4 | (DE "PREMATURE labor" OR DE "PREMATURE infants" OR DE "BIRTH weight" OR DE "LOW birth weight" OR DE "SMALL for gestational age" OR DE "VERY low birth weight") | 52,396 |
| 5 | S1 OR S2 | 286,728 |
| 6 | S3 OR S4 | 426,384 |
| 7 | S5 AND S6 | 844 |

### Environment Complete (EBSCOhost)

| 1 | TI ( (Hurricane* OR Cyclone* OR Flood* OR Storm* OR Rainstorm* OR Thunderstorm* OR Superstorm* OR Tempest* OR “Heavy Rain*” OR Monsoon* OR Typhoon* OR “Tidal wave*” OR tidalwave* OR inundation* OR tsunami* OR meteotsunami* OR deluge*) ) OR AB ( (Hurricane* OR Cyclone* OR Flood* OR Storm* OR Rainstorm* OR Thunderstorm* OR Superstorm* OR Tempest* OR “Heavy Rain*” OR Monsoon* OR Typhoon* OR “Tidal wave*” OR tidalwave* OR inundation* OR tsunami* OR meteotsunami* OR deluge*) ) OR KW ( (Hurricane* OR Cyclone* OR Flood* OR Storm* OR Rainstorm* OR Thunderstorm* OR Superstorm* OR Tempest* OR “Heavy Rain*” OR Monsoon* OR Typhoon* OR “Tidal wave*” OR tidalwave* OR inundation* OR tsunami* OR meteotsunami* OR deluge*) ) | 157,808 |
| --- | --- | --- |
| 2 | (DE "FLOODS" OR DE "FLOOD damage" OR DE "STORM surges" OR DE "RAINSTORMS" OR DE "THUNDERSTORMS" OR DE "TYPHOONS" OR DE "CYCLONES" OR DE "HURRICANES" OR DE "TROPICAL cyclones" OR DE "TYPHOONS" OR DE "HURRICANES" OR DE "TSUNAMIS" OR DE "METEOTSUNAMIS") | 36,412 |
| 3 | S1 OR S2 | 159,376 |
| 4 | TI ( (Preterm* OR Birth* OR Lowbirth* OR Gestation* OR ((Pregnan* OR Prenatal* OR pre-natal* OR Perinatal* OR peri-natal* OR “in utero*”) N15 (Time OR Timing OR Duration OR Length OR Span OR interval* OR period* OR weight*))) ) OR AB ( (Preterm* OR Birth* OR Lowbirth* OR Gestation* OR ((Pregnan* OR Prenatal* OR pre-natal* OR Perinatal* OR peri-natal* OR “in utero*”) N15 (Time OR Timing OR Duration OR Length OR Span OR interval* OR period* OR weight*))) ) OR KW ( (Preterm* OR Birth* OR Lowbirth* OR Gestation* OR ((Pregnan* OR Prenatal* OR pre-natal* OR Perinatal* OR peri-natal* OR “in utero*”) N15 (Time OR Timing OR Duration OR Length OR Span OR interval* OR period* OR weight*))) ) | 38,085 |
| 5 | S3 AND S4 | 178 |

### Scopus

TITLE-ABS-KEY ( ( hurricane* OR cyclone* OR flood* OR storm* OR rainstorm* OR thunderstorm* OR superstorm* OR tempest* OR "Heavy Rain*" OR monsoon* OR typhoon* OR "Tidal wave*" OR tidalwave* OR inundation* OR tsunami* OR meteotsunami* OR deluge* ) AND ( preterm* OR birth* OR lowbirth* OR gestation* OR ( ( pregnan* OR prenatal* OR pre-natal* OR perinatal* OR peri-natal* OR "in utero*" ) W/15 ( time OR timing OR duration OR length OR span OR interval* OR period* OR weight* ) ) ) )

### Web of Science Core Collection (Web of Science)

TS=((Hurricane* OR Cyclone* OR Flood* OR Storm* OR Rainstorm* OR Thunderstorm* OR Superstorm* OR Tempest* OR “Heavy Rain*” OR Monsoon* OR Typhoon* OR “Tidal wave*” OR tidalwave* OR inundation* OR tsunami* OR meteotsunami* OR deluge*) AND (Preterm* OR Birth* OR Lowbirth* OR Gestation* OR ((Pregnan* OR Prenatal* OR pre-natal* OR Perinatal* OR peri-natal* OR “in utero*”) N15 (Time OR Timing OR Duration OR Length OR Span OR interval* OR period* OR weight*))))

### CAB abstracts (CABI)

*Note: the CABI interface search syntax was updated between April 2024 and February 2025.*

In both searches, used AND operator in place of adjacency/proximity that was used in other searches.

**February 2025**

| 1 | (Title:((Hurricane* OR Cyclone* OR Flood* OR Storm* OR Rainstorm* OR Thunderstorm* OR Superstorm* OR Tempest* OR "Heavy Rain*" OR Monsoon* OR Typhoon* OR "Tidal wave*" OR tidalwave* OR inundation* OR tsunami* OR meteotsunami* OR deluge*)) OR ab:((Hurricane* OR Cyclone* OR Flood* OR Storm* OR Rainstorm* OR Thunderstorm* OR Superstorm* OR Tempest* OR "Heavy Rain*" OR Monsoon* OR Typhoon* OR "Tidal wave*" OR tidalwave* OR inundation* OR tsunami* OR meteotsunami* OR deluge*)) OR id:((Hurricane* OR Cyclone* OR Flood* OR Storm* OR Rainstorm* OR Thunderstorm* OR Superstorm* OR Tempest* OR "Heavy Rain*" OR Monsoon* OR Typhoon* OR "Tidal wave*" OR tidalwave* OR inundation* OR tsunami* OR meteotsunami* OR deluge*)) OR indexingterm:("hurricanes" OR "flooding" OR "floods" OR "Storms" OR "cyclones" OR "tempests" OR "Monsoons" OR "Typhoons" OR "tidal waves" OR "tsunamis")) AND (Title:((Preterm* OR Birth* OR Lowbirth* OR Gestation* OR ((Pregnan* OR Prenatal* OR pre-natal* OR Perinatal* OR peri-natal* OR "in utero*") |
| --- | --- |
| 2 | (Time OR Timing OR Duration OR Length OR Span OR interval* OR period* OR weight*)))) OR ab:((Preterm* OR Birth* OR Lowbirth* OR Gestation* OR ((Pregnan* OR Prenatal* OR pre-natal* OR Perinatal* OR peri-natal* OR "in utero*") AND (Time OR Timing OR Duration OR Length OR Span OR interval* OR period* OR weight*)))) OR id:((Preterm* OR Birth* OR Lowbirth* OR Gestation* OR ((Pregnan* OR Prenatal* OR pre-natal* OR Perinatal* OR peri-natal* OR "in utero*") AND (Time OR Timing OR Duration OR Length OR Span OR interval* OR period* OR weight*)))) OR indexingterm:("birth weight" OR "preterm infants" OR "gestation period" OR "gestation length" OR "perinatal period")) |
| 3 | 1 AND 2 |

**April 2024**

| 1 | Title:((Preterm* OR Birth* OR Lowbirth* OR Gestation* OR ((Pregnan* OR Prenatal* OR pre-natal* OR Perinatal* OR peri-natal* OR "in utero*") AND (Time OR Timing OR Duration OR Length OR Span OR interval* OR period* OR weight*)))) OR ab:((Preterm* OR Birth* OR Lowbirth* OR Gestation* OR ((Pregnan* OR Prenatal* OR pre-natal* OR Perinatal* OR peri-natal* OR "in utero*") AND (Time OR Timing OR Duration OR Length OR Span OR interval* OR period* OR weight*)))) OR Keyword:((Preterm* OR Birth* OR Lowbirth* OR Gestation* OR ((Pregnan* OR Prenatal* OR pre-natal* OR Perinatal* OR peri-natal* OR "in utero*") AND (Time OR Timing OR Duration OR Length OR Span OR interval* OR period* OR weight*)))) OR indexingterm:(("birth weight" OR "preterm infants" OR "gestation period" OR "gestation length" OR "perinatal period")) |
| --- | --- |
| 2 | Title:((Hurricane* OR Cyclone* OR Flood* OR Storm* OR Rainstorm* OR Thunderstorm* OR Superstorm* OR Tempest* OR "Heavy Rain*" OR Monsoon* OR Typhoon* OR "Tidal wave*" OR tidalwave* OR inundation* OR tsunami* OR meteotsunami* OR deluge*)) OR ab:((Hurricane* OR Cyclone* OR Flood* OR Storm* OR Rainstorm* OR Thunderstorm* OR Superstorm* OR Tempest* OR "Heavy Rain*" OR Monsoon* OR Typhoon* OR "Tidal wave*" OR tidalwave* OR inundation* OR tsunami* OR meteotsunami* OR deluge*)) OR Keyword:((Hurricane* OR Cyclone* OR Flood* OR Storm* OR Rainstorm* OR Thunderstorm* OR Superstorm* OR Tempest* OR "Heavy Rain*" OR Monsoon* OR Typhoon* OR "Tidal wave*" OR tidalwave* OR inundation* OR tsunami* OR meteotsunami* OR deluge*)) OR indexingterm:(("hurricanes" OR "flooding" OR "floods" OR "Storms" OR "cyclones" OR "tempests" OR "Monsoons" OR "Typhoons" OR "tidal waves" OR "tsunamis")) |
| 3 | 1 AND 2 |

### Environmental Science Database & Index (ProQuest)

| S1 | title((Hurricane* OR Cyclone* OR Flood* OR Storm* OR Rainstorm* OR Thunderstorm* OR Superstorm* OR Tempest* OR “Heavy Rain*” OR Monsoon* OR Typhoon* OR “Tidal wave*” OR tidalwave* OR inundation* OR tsunami* OR meteotsunami* OR deluge*) ) OR abstract((Hurricane* OR Cyclone* OR Flood* OR Storm* OR Rainstorm* OR Thunderstorm* OR Superstorm* OR Tempest* OR “Heavy Rain*” OR Monsoon* OR Typhoon* OR “Tidal wave*” OR tidalwave* OR inundation* OR tsunami* OR meteotsunami* OR deluge*) ) OR if((Hurricane* OR Cyclone* OR Flood* OR Storm* OR Rainstorm* OR Thunderstorm* OR Superstorm* OR Tempest* OR “Heavy Rain*” OR Monsoon* OR Typhoon* OR “Tidal wave*” OR tidalwave* OR inundation* OR tsunami* OR meteotsunami* OR deluge*)) | 357,531 |
| --- | --- | --- |
| S2 | (MAINSUBJECT.EXACT("Floods") OR MAINSUBJECT.EXACT("Flooded areas") OR MAINSUBJECT.EXACT("Hurricanes") OR MAINSUBJECT.EXACT("Stormwater") OR MAINSUBJECT.EXACT("Cyclones") OR MAINSUBJECT.EXACT("Tropical cyclones") OR MAINSUBJECT.EXACT("Tidal waves") OR MAINSUBJECT.EXACT("Thunderstorms") OR MAINSUBJECT.EXACT("Rainstorms") OR MAINSUBJECT.EXACT("Tsunamis") OR MAINSUBJECT.EXACT("Typhoons") OR MAINSUBJECT.EXACT("Storm tides") OR MAINSUBJECT.EXACT("Tidal waves")) | 131,655 |
| S3 | title((Preterm* OR Birth* OR Lowbirth* OR Gestation* OR ((Pregnan* OR Prenatal* OR pre-natal* OR Perinatal* OR peri-natal* OR “in utero*”) NEAR/15 (Time OR Timing OR Duration OR Length OR Span OR interval* OR period* OR weight*)))) OR abstract((Preterm* OR Birth* OR Lowbirth* OR Gestation* OR ((Pregnan* OR Prenatal* OR pre-natal* OR Perinatal* OR peri-natal* OR “in utero*”) NEAR/15 (Time OR Timing OR Duration OR Length OR Span OR interval* OR period* OR weight*)))) OR if((Preterm* OR Birth* OR Lowbirth* OR Gestation* OR ((Pregnan* OR Prenatal* OR pre-natal* OR Perinatal* OR peri-natal* OR “in utero*”) NEAR/15 (Time OR Timing OR Duration OR Length OR Span OR interval* OR period* OR weight*)))) | 239,914 |
| S4 | (MAINSUBJECT.EXACT("Birth weight") OR MAINSUBJECT.EXACT("Small for gestational age") OR MAINSUBJECT.EXACT("Low birth weight") OR MAINSUBJECT.EXACT("Premature birth")) | 29,821 |
| S5 | [S1] OR [S2] | 378,787 |
| S6 | [S3] OR [S4] | 244,191 |
| S7 | [S5] AND [S6] | 572 |

### PubMed

Used AND operator in place of adjacency/proximity that was used in other searches.

| 1 | (Hurricane*[Title/Abstract] OR Cyclone*[Title/Abstract] OR Flood*[Title/Abstract] OR Storm*[Title/Abstract] OR Rainstorm*[Title/Abstract] OR Thunderstorm*[Title/Abstract] OR Superstorm*[Title/Abstract] OR Tempest*[Title/Abstract] OR "Heavy Rain*"[Title/Abstract] OR Monsoon*[Title/Abstract] OR Typhoon*[Title/Abstract] OR "Tidal wave*"[Title/Abstract] OR tidalwave*[Title/Abstract] OR inundation*[Title/Abstract] OR tsunami*[Title/Abstract] OR meteotsunami*[Title/Abstract] OR deluge*[Title/Abstract]) | 65,184 |
| --- | --- | --- |
| 2 | ("Floods"[Mesh] OR "Cyclonic Storms"[Mesh] OR "Tidal Waves"[Mesh] OR "Tsunamis"[Mesh]) | 8,486 |
| 3 | #1 OR #2 | 66,319 |
| 4 | (Preterm*[Title/Abstract] OR Birth*[Title/Abstract] OR Lowbirth*[Title/Abstract] OR Gestation*[Title/Abstract] OR ((Pregnan*[Title/Abstract] OR Prenatal*[Title/Abstract] OR pre-natal*[Title/Abstract] OR Perinatal*[Title/Abstract] OR peri-natal*[Title/Abstract] OR "in utero*"[Title/Abstract]) AND (Time[Title/Abstract] OR Timing[Title/Abstract] OR Duration[Title/Abstract] OR Length[Title/Abstract] OR Span[Title/Abstract] OR interval*[Title/Abstract] OR period*[Title/Abstract] OR weight*[Title/Abstract]))) | 752,095 |
| 5 | ("Term Birth"[Mesh] OR "Premature Birth"[Mesh] OR "Obstetric Labor, Premature"[Mesh] OR "Birth Weight"[Mesh]) | 82,104 |
| 6 | #4 OR #5 | 768,319 |
| 7 | #3 AND #6 | 726 |
