# Supplementary material for: The effect of flooding on low birthweight and preterm birth: a systematic review and meta-analysis
Source: BMC Public Health. 2026 Mar 5;26:800. doi: 10.1186/s12889-026-26521-2 (PMC12961868; doi:10.1186/s12889-026-26521-2)
Supplement: Supplementary file 5 — Additional file 5: Subgroup forest plots for covariates and U.S. only studies. [file 12889_2026_26521_MOESM5_ESM.docx]

**Additional File 5**

| 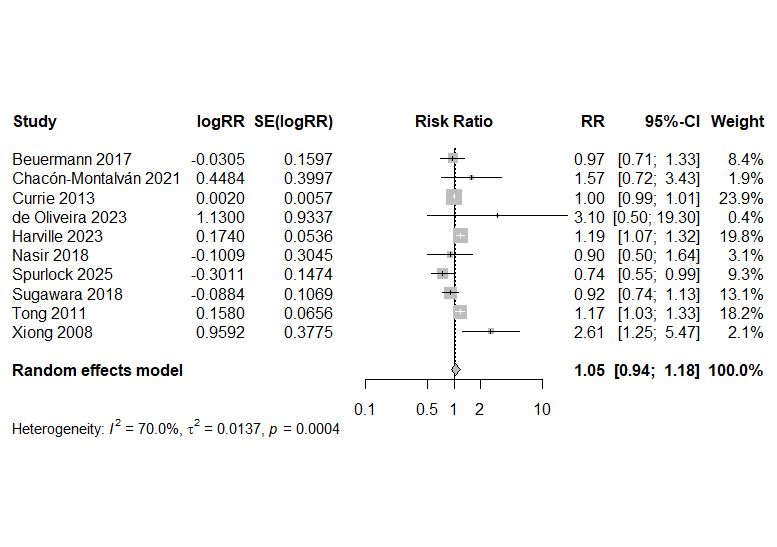 | 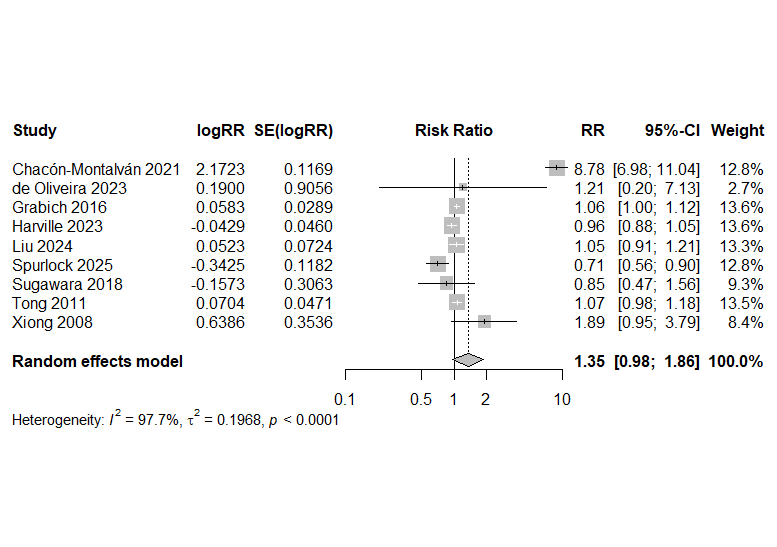 |
| --- | --- |

Figure S1: Subgroup analysis forest plots by adjusted covariates (maternal age and maternal education) for Low Birth Weight (A) and Preterm Birth (B).

| 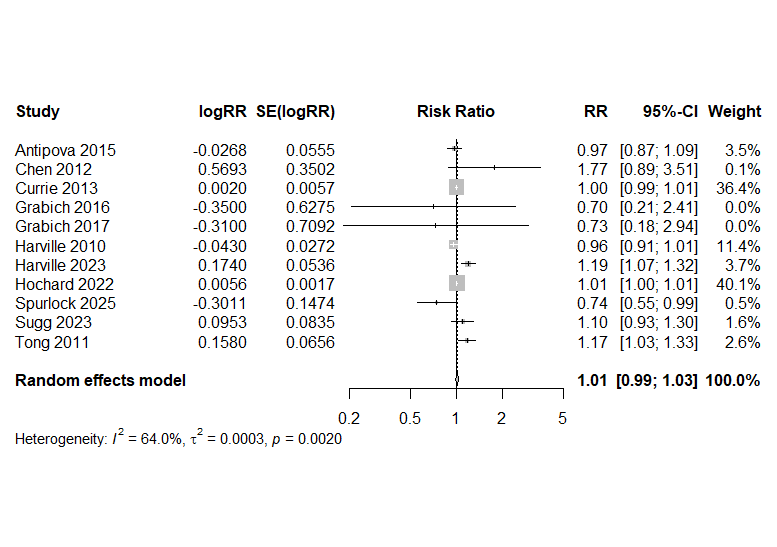 | 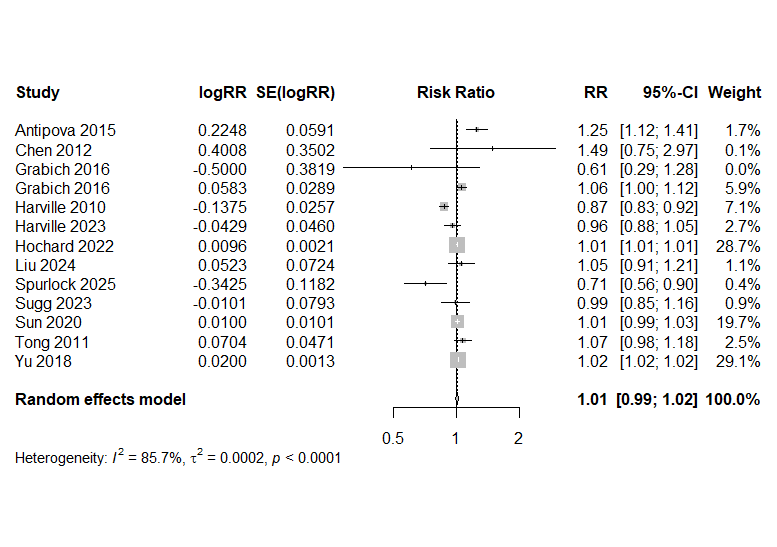 |
| --- | --- |

Figure S2: Subgroup analysis forest plots for studies in the United States for Low Birth Weight (A) and Preterm Birth (B).
